# SNAP-tag2 for faster and brighter protein labeling

**DOI:** 10.1038/s41589-025-01942-z

**Published:** 2025-07-03

**Authors:** Stefanie Kühn, Veselin Nasufovic, Jonas Wilhelm, Julian Kompa, Eline M. F. de Lange, Yin-Hsi Lin, Cornelia Egoldt, Jonas Fischer, Artem Lennoi, Miroslaw Tarnawski, Jochen Reinstein, Rifka Vlijm, Julien Hiblot, Kai Johnsson

**Affiliations:** 1 Department of Chemical Biology, Max Planck Institute for Medical Research, Heidelberg, Germany; 2 Molecular Biophysics, Zernike Institute for Advanced Materials, University of Groningen, Groningen, The Netherlands; 3 Molecular Cell Biology, Groningen Biomolecular Sciences and Biotechnology Institute, University of Groningen, Groningen, The Netherlands; 4 Protein Expression and Characterization Facility, Max Planck Institute for Medical Research, Heidelberg, Germany; 5 Department of Biomolecular Mechanisms, Max Planck Institute for Medical Research, Heidelberg, Germany; 6 Institute of Chemical Sciences and Engineering, École Polytechnique Fédérale de Lausanne (EPFL), Lausanne, Switzerland

**Keywords:** Chemical tools, Protein design, Imaging

## Abstract

SNAP-tag is a powerful tool for labeling proteins with synthetic fluorophores in bioimaging. However, its utility in live-cell applications can be constrained by its relatively slow labeling kinetics and the limited cell permeability of its substrates. Here, we introduce improved labeling substrates and an engineered SNAP-tag for faster labeling in vitro and in live cells. SNAP-tag2 presents a second-order rate constant with rhodamine substrates that approaches 10^7^ s^−1^ M^−1^, a 100-fold improvement over the corresponding SNAP-tag–substrate pairs. When labeled with highly fluorogenic dyes, SNAP-tag2 also shows a fivefold increase in fluorescence brightness relative to currently used SNAP-tag. The increased labeling kinetics and brightness of SNAP-tag2 translate into greatly improved performance in various live-cell (super-resolution) imaging applications.

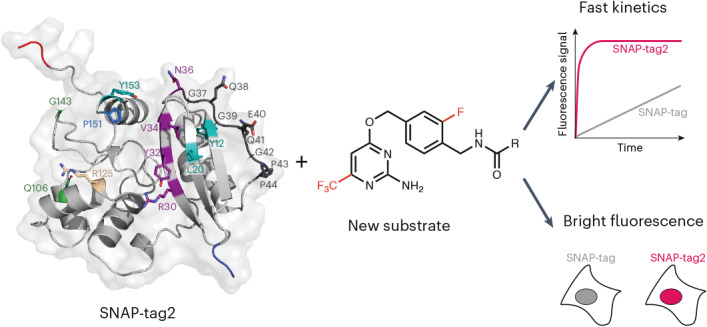

## Abstract (web summary)

SNAP-tag is a widespread tool for labeling protein for bioimaging. Now, Kühn et al. report SNAP-tag2 with increased labeling kinetics and brightness, which translates into a better performance in live-cell super-resolution imaging.

## Main

Self-labeling protein tags (SLPs) can be specifically and covalently labeled with synthetic probes in vitro and in live cells^1,2^. Popular examples of such tags are SNAP-tag^3,4^, HaloTag7 (ref. ^5^) and, to a lesser extent, CLIP-tag^6^. Their main field of use has been in bioimaging, where these tags offer the opportunity to attach bright and photostable synthetic fluorophores^7^ to proteins of interest. The development of fluorogenic probes for protein labeling (that is, probes that only become highly fluorescent upon binding to the protein of interest) has further facilitated the use of SLPs in live-cell bioimaging as it decreases background signal from unbound dye^8,9^.

HaloTag7 is at present the most popular SLP for live-cell imaging. HaloTag7 was engineered to react fast and efficiently with chloroalkane (CA)–rhodamine substrates^10^ (Supplementary Fig. 1). Rhodamines are well-suited synthetic fluorophores for live-cell imaging as they cover a broad spectral range, possess excellent spectroscopic properties^11^, can be fluorogenic^8,12^ and are permeable in live cells^13,14^. The fluorogenicity and permeability of rhodamines can probably be attributed to the reversible spirocyclization of the zwitterionic, fluorescent rhodamine to a nonpolar and nonfluorescent spirolactone^9,14,15^. For selected CA–rhodamines, labeling kinetics of HaloTag7 are close to the diffusion limit. The high reactivity of HaloTag7 toward CA–rhodamines is at least partially because of specific interactions of the protein surface with the rhodamine dye^16^. The combination of the high labeling velocity of HaloTag7 with CA–rhodamines, their relatively good permeability and the excellent performance of these dyes in various microscopy applications have made the approach a powerful tool in bioimaging^17^.

**Fig. 1:**
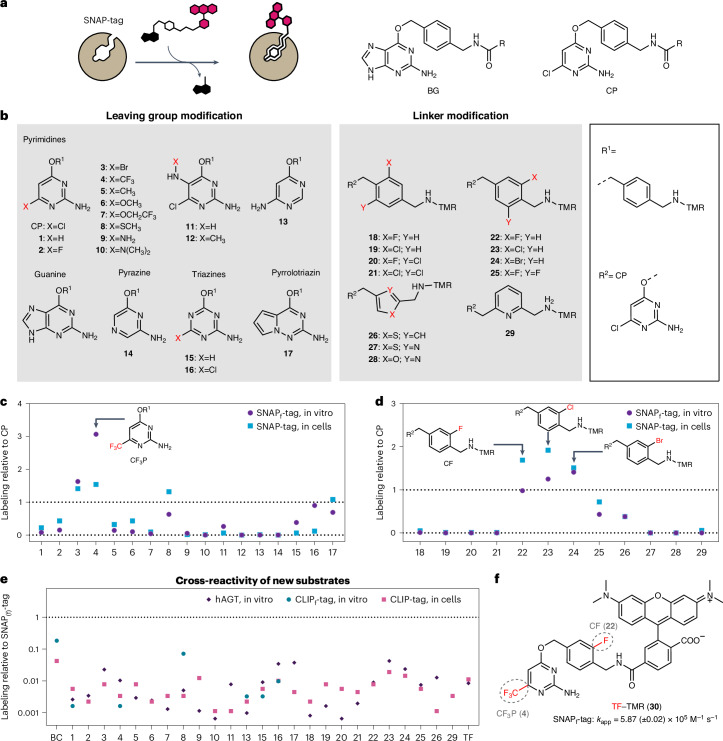
SNAP-tag substrate screening for more efficient labeling in vitro and in live mammalian cells. **a**, Scheme of SNAP-tag labeling reaction with fluorophore substrates. The chemical structures of SNAP-tag substrates BG and CP are shown on the right. R represents the functional moiety to be linked to SNAP-tag. **b**, Chemical structures of modified SNAP-tag substrates divided into two groups: leaving group modification and linker modification. Modified leaving groups were linked to TMR over linker R^1^. Modified linkers coupled to TMR were tested on the CP (R^2^) scaffold. **c**,**d**, Comparison of substrates **1**–**17** (**c**) and substrates **18**–**29** (**d**) relative to SNAP_(f)_-tag labeling with CP–TMR regarding their in vitro labeling kinetics and performance in live-cell labeling. In vitro labeling kinetics of SNAP_f_-tag with different substrates were measured recording FP traces over time. The *k*_app_ was calculated (Supplementary Table 1) and normalized to the *k*_app_ of SNAP_f_-tag with CP–TMR. Live-cell performance of developed substrates was tested by labeling of U2OS cells stably expressing an mEGFP–SNAP-tag fusion protein with TMR substrates at 100 nM for 2 h. Cells were washed and analyzed by flow cytometry. Fluorescence intensity ratios of TMR to mEGFP were calculated (Supplementary Table 1) and normalized to the ratio obtained for SNAP-tag with CP–TMR. Leaving group substrate **4** and linker substrates **22**, **23** and **24** showed the most promising results relative to CP–TMR. **e**, Reactivity of selected SNAP-tag substrates with hAGT and CLIP_(f)_-tag compared to SNAP_(f)_-tag labeling with CP–TMR. Experiments were conducted as previously described. **f**, Chemical structure of TF–TMR (**30**) found through the combination of leaving group **4** and linker **22**. The *k*_app_ of SNAP_f_-tag labeling with TF–TMR is depicted below.

SNAP-tag undergoes a nucleophilic substitution reaction with *O*^6^-benzylguanine (BG) or chloropyrimidine (CP) derivatives, in which the benzyl group of the substrate is irreversibly transferred to an active site reactive cysteine residue (Fig. 1a). SNAP-tag is a highly engineered human *O*^6^-alkylguanine-DNA alkyltransferase (hAGT) that was optimized for reaction with BG derivatives^3,18–20^. While BG derivatives show faster labeling kinetics with SNAP-tag than the corresponding CP derivatives^16^, the less polar CP derivatives generally perform better in live-cell imaging^21^. However, compared to HaloTag7, SNAP-tag labeling kinetics with rhodamine-based substrates are in general at least two orders of magnitude slower^16^. Additionally, HaloTag7 shows higher fluorogenicity with rhodamines than SNAP-tag^9,22^. This is presumably because of specific interactions between the fluorophore and the HaloTag7 surface, which promote an equilibrium shift from the nonfluorescent spirolactone to the fluorescent zwitterionic state^23^. No such specific interactions between SNAP-tag and rhodamine-based fluorophores have been identified^16^.

The limited cell permeability of the SNAP-tag substrate and the reduced labeling kinetics and fluorogenicity of the SNAP-tag can impede its use in certain live-cell imaging applications. Here, we present SNAP-tag2, a highly engineered SNAP-tag mutant that rapidly reacts with optimized pyrimidine-based substrates for live-cell applications, and demonstrate the effectiveness of SNAP-tag2 and its substrates in a range of bioimaging applications.

## Results

### Improved SNAP-tag substrates

**Extended Data Fig. 1:**
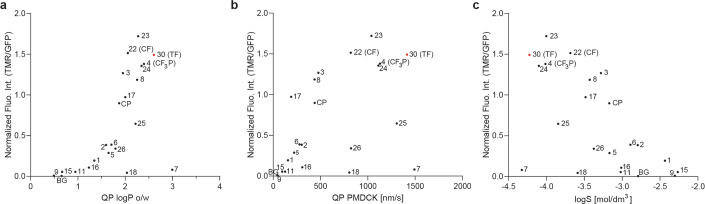
Correlation between pharmacokinetic properties and live cell performance of selected substrates. QikProp (QP) Descriptors were calculated for non-fluorescent acetylated substrates using Schrödinger Maestro 12.3 software, regarding their: **a**, octanol/water partition coefficient (QP logP o/w), **b**, apparent MDCK cell permeability (QP PMDCK) and **c**, aqueous solubility (logS) and correlated to the experimentally determined performance of their corresponding TMR-derivatives in live cells. Computed parameters of the acetylated substrates can be found in Supplementary Table 2.

**Extended Data Fig. 2:**
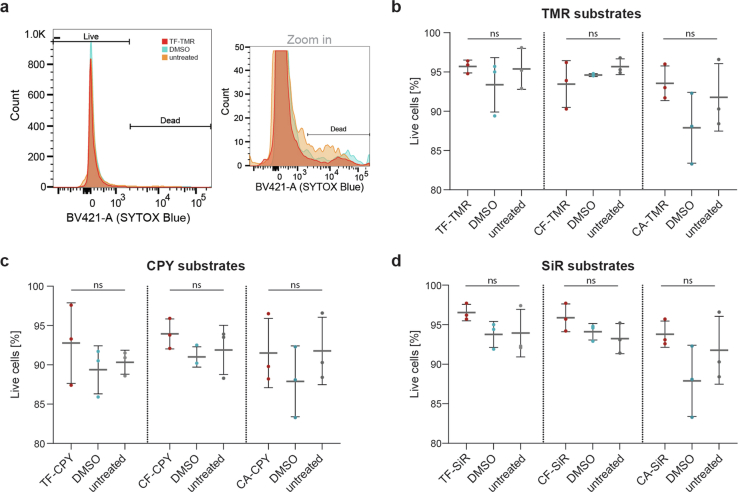
Statistical analysis of cell viability using flow cytometry. U2OS cells were incubated either with TF-/CF-/CA-substrates [1 µM], DMSO (1 % v/v) or remained untreated. After 1 h incubation at 37 °C, both dead cells (supernatant) and live cells (detached with trypsin) were collected. SYTOX Blue dead cell stain [1 µM] was added and the cells were subsequently analyzed by flow cytometry. Experiment was conducted in technical triplicates. **a**, Flow cytometry histograms of cells stained with SYTOX Blue, showing the gating strategy for live and dead cell events. **b**–**d** Dot plots showing the percentage of live cell events for **b** TMR-substrates, **c** CPY-substrates and d SiR-substrates in comparison to DMSO and untreated control samples. Each dot represents one replicate. The mean percentage of live cells from each triplicate is indicated as a black horizontal line. Error bars represent the s.d. The differences in cell viability of treated and untreated cells were not statistically significant (two-tailed unpaired t-test with Welch’s correction, p > 0.05, ns). Novel TF- and CF-substrates do not influence cell viability. Source data

To improve the performance of SNAP-tag in live-cell imaging, we decided to first develop different substrate scaffolds for higher intrinsic reactivity in vitro and improved live-cell compatibility, followed by engineering of SNAP-tag for faster reaction kinetics and enhanced fluorescence brightness with these substrates. SNAP-tag substrates can be divided into two parts: the leaving group and the linker that connects the synthetic probe to the reactive cysteine (Fig. 1b). We synthesized 17 different heterocycles connected through a benzyl linker to tetramethylrhodamine (TMR) and measured their labeling kinetics in vitro with SNAP_f_-tag, a single-point mutant of SNAP-tag with increased labeling kinetics^16,24^, as well as their performance of labeling SNAP-tag in a cellular assay (Fig. 1c,d and Supplementary Table 1). The selection of heterocycles was based on previous observations that pyrimidines can be good substrates for SNAP-tag^21,25^ but also comprised pyrazines, triazines and a pyrrolotriazine. Of these compounds, the trifluoromethyl-substituted pyrimidine **4** (CF_3_P) showed the best results (Fig. 1c), with a roughly 3-fold increase in labeling kinetics in vitro and 1.5-fold higher fluorescence labeling in live cells. Additionally, we investigated 12 different linkers connecting CP as the leaving group and TMR as the fluorescent probe. The fluoro and chloro derivatives **22** and **23** showed 2-fold increased fluorescence labeling in the cellular assay (Fig. 1d). However, the chloro analog **23** showed increased reactivity against parental hAGT (Fig. 1e). Therefore, to reduce the risk of unwanted background reactions, it was excluded. We furthermore computed the octanol–water partition coefficient (QP logP o/w), aqueous solubility (QP logS) and apparent MDCK permeability (QP PMDCK) of the various substrates as their nonfluorescent *N*-acetylated derivatives (Extended Data Fig. 1 and Supplementary Table 2). The substrates with the highest predicted permeability and logP o/w, which additionally showed fast labeling of SNAP_f_-tag in vitro when coupled to TMR, also performed best in live-cell fluorescence labeling experiments (Extended Data Fig. 1). An exception was compound **7**, for which high permeability was predicted but low efficiency for labeling of SNAP-tag in live cells was observed (Fig. 1c). Combining the features of the best-performing substrates **4** (CF_3_P) and **22** (CF) yielded trifluoromethyl fluorobenzyl pyrimidine **30** (TF), which, as a TMR derivative (Fig. 1f), showed an apparent second-order rate constant (*k*_app_) of 5.87 (±0.02) × 10^5^ M^−1^ s^−1^ for the labeling of SNAP_f_-tag (Supplementary Fig. 2), corresponding to 3.9-fold faster labeling kinetics than its reaction with CP–TMR (*k*_app_ = 1.51 (±0.01) × 10^5^ M^−1^ s^−1^)^16^. Moreover, TF–TMR showed ~1.7-fold higher fluorescence labeling than CP–TMR in live cells (Supplementary Fig. 3). We further demonstrated that the optimized substrates do not significantly influence cell viability, even at substrate concentrations higher than used in labeling (Extended Data Fig. 2).

### Engineering of SNAP-tag protein

**Fig. 2:**
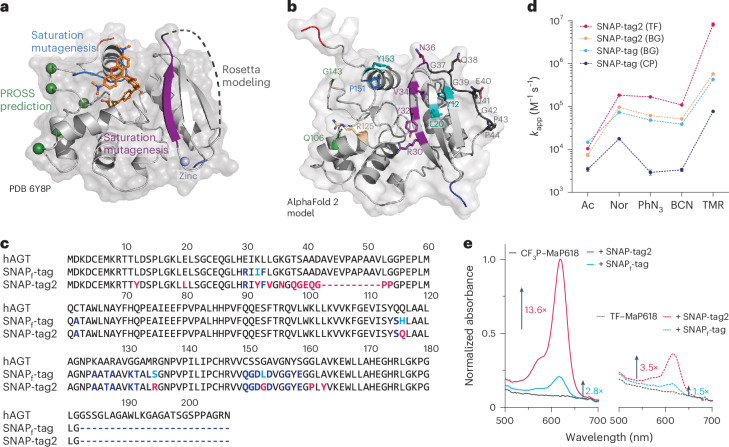
SNAP-tag engineering aiming for faster and brighter fluorescence labeling. **a**, Crystal structure of SNAP-tag labeled with TMR (PDB 6Y8P) with engineered regions highlighted. SNAP-tag is represented as a light-gray cartoon and the TMR ligand is represented as sticks. Saturation mutagenesis libraries were constructed on the C-terminal loop region (residues 155–161) and the β-strand proximal to the active site (residues 29–36), highlighted in blue and violet, respectively. Substitutions predicted by PROSS^26^ to increase the protein thermal stability are highlighted as green spheres. The unstructured region in SNAP-tag (residues 37–55) highlighted as a gray dotted line was redesigned using RosettaRemodel^27^. Termini are highlighted in blue (N terminus) and red (C terminus) and the coordinated zinc ion is illustrated as a light-blue sphere. **b**, AlphaFold 2 (ref. ^36^) model of SNAP-tag2. Introduced substitutions are highlighted as sticks and color-coded on the basis of the engineering rationale. Green, PROSS^26^ prediction; marine blue and deep purple, saturation mutagenesis libraries; wheat, rational design; teal, sDMSL; dark gray, Rosetta-modeled loop. Termini are highlighted in blue (N terminus) and red (C terminus). **c**, Sequence alignment of hAGT, SNAP_f_-tag and SNAP-tag2. Dark blue, common differences of SNAP_f_-tag and SNAP-tag2 versus hAGT; turquoise, unique differences SNAP_f_-tag versus hAGT; pink, unique differences SNAP-tag2 versus hAGT and/or SNAP_f_-tag. Amino acid deletions are presented as dotted lines. **d**, Comparison of labeling kinetics between SNAP-tag2 and SNAP-tag. The *k*_app_ of SNAP-tag2 with TF substrates demonstrate one to two orders of magnitude faster labeling kinetics compared to the reaction of parental SNAP-tag with CP substrates. Labeling kinetics of SNAP-tag2 with BG substrates remained unchanged compared to SNAP-tag labeling. CP and BG results for SNAP-tag labeling were taken from Wilhelm et al.^16^. Abbreviations: Ac, acetate; BCN, biscyclononyne; Nor, (1*S*,4*S*)-5-methylbicyclo[2.2.1]hept-2-ene (norbornene); PhN_3_, phenylazide. **e**, Normalized absorbance spectra of fluorogenic CF_3_P–MaP618 and TF–MaP618 substrates (15 µM) in the presence or absence of SNAP-tag2/SNAP_f_-tag protein (30 µM). Fold changes are referred to the absorbance of the dye only. SNAP-tag2 shows approximately 4.9-fold and 2.3-fold increases in absorbance of fluorogenic substrates for CF_3_P–MaP618 and TF–MaP618, respectively, compared to SNAP_f_-tag. Source data

In addition to substrate optimization, we used protein engineering to increase the reactivity of SNAP-tag toward pyrimidine-based substrates and its brightness when labeled with fluorogenic rhodamines such as MaP618 (ref. ^9^). Specifically, we used mutagenesis of selected residues, computational design and directed evolution to identify mutants with increased reactivity and fluorogenicity (Fig. 2 and Supplementary Fig. 4). The engineering was guided by the available X-ray structures of SNAP-tag in its apo (Protein Data Bank (PDB) 3KZY), benzylated (PDB 3L00), BG-bound (PDB 3KZZ)^4^ and TMR-labeled (PDB 6Y8P)^16^ states and by past engineering efforts^3,18,19,24^ (Fig. 2a). We modified S135 to arginine, assuming it might favorably interact with the carboxylate of rhodamine substrates. We also used the computational method PROSS^26^ to identify amino acid substitutions that could increase the thermal stability of SNAP-tag (Fig. 2a). Additionally, the N-terminal domain of SNAP-tag comprises a long, unstructured region (residues 37–54; Fig. 2a–c), which might compromise the protein’s stability. To address this, we used RosettaRemodel^27^ to design a short eight-residue loop as a replacement for this unstructured region (Fig. 2b,c). For directed evolution experiments, protein libraries of two distinct regions in close proximity to the substrate-binding site were created by saturation mutagenesis (Fig. 2a): the β-strand proximal to the active site (residues 29–36) and the active site loop (residues 155–161). Lastly, a synthetic deep mutational scanning library (sDMSL)^28^ was screened, in which each residue in SNAP-tag was individually replaced by all other 19 amino acids, excluding cysteines but including deletions. Deep mutational scanning allows systematically studying the impact of individual variations in the context of SNAP-tag and identifying the best amino acids at each position. Libraries were screened for enhanced labeling kinetics with the developed TMR substrates and/or increased fluorescence intensity of MaP618 derivatives (Supplementary Fig. 1) using yeast surface display (YSD) combined with fluorescence-activated cell sorting (Supplementary Fig. 4). These engineering efforts led to the identification of SNAP-tag2, which carries 11 substitutions and a replacement of 18 amino acids (residues 37–54) by a peptide of eight amino acids (Fig. 2b,c), without destabilizing the protein (melting temperature (*T*_m_) of around 65 °C; Supplementary Fig. 5). The *k*_app_ for its reaction with TF–TMR was 8.22 (±0.79) × 10^6^ M^−1^ s^−1^ (Table 1, Supplementary Fig. 6 and Supplementary Tables 3 and 4), which was about 100-fold faster than the reaction of SNAP-tag with CP–TMR (Fig. 2d) and close to that for the reaction of HaloTag7 with CA–TMR (*k*_app_ = 1.88 (±0.01) × 10^7^ M^−1^ s^−1^)^16^. SNAP-tag2 also showed increased reaction kinetics with CP-based and CF-based substrates and reached its fastest labeling reaction with TF–CPY in vitro with a *k*_app_ of 1.04 (±0.12) × 10^7^ M^−1^ s^−1^ (Table 1, Supplementary Figs. 7–9 and Supplementary Tables 3 and 4). Furthermore, SNAP-tag2 showed increased reactivity toward nonfluorescent probes (Fig. 2d, Supplementary Fig. 10 and Supplementary Table 5). The in vitro labeling kinetics of SNAP-tag2 with BG substrates were determined to be very similar to those of SNAP-tag with these substrates (Fig. 2d and Supplementary Figs. 10 and 11). The extinction coefficients and quantum yields (QYs) of SNAP-tag2 labeled with different rhodamines were similar to those of its predecessor, except for very fluorogenic MaP618 substrates (Supplementary Table 6). For these, SNAP-tag2 showed a significantly higher absorbance than SNAP_f_-tag, (Fig. 2e), indicating that SNAP-tag2 might have a higher capacity to shift the dye toward its zwitterionic, fluorescent state.

**Table 1:** The *k*_app_ of SNAP-tag2 labeling with different TMR and CPY substrates

TMR substrate	*k*_app_ (±s.d.) (M^−1^ s^−1^)	CPY substrate	*k*_app_ (±s.d.) (M^−1^ s^−1^)
TF–TMR	8.22 (±0.79) × 10^6^	TF–CPY	1.04 (±0.12) × 10^7^
CF–TMR	6.21 (±3.65) × 10^6^	CF–CPY	3.62 (±0.73) × 10^6^
CP–TMR	7.82 (±2.78) × 10^6^	CP–CPY	5.29 (±1.62) × 10^6^

Values represent the average *k*_app_ values calculated from experimental replicates (Supplementary Table 3).

### Performance of SNAP-tag2 in live-cell imaging

**Fig. 3:**
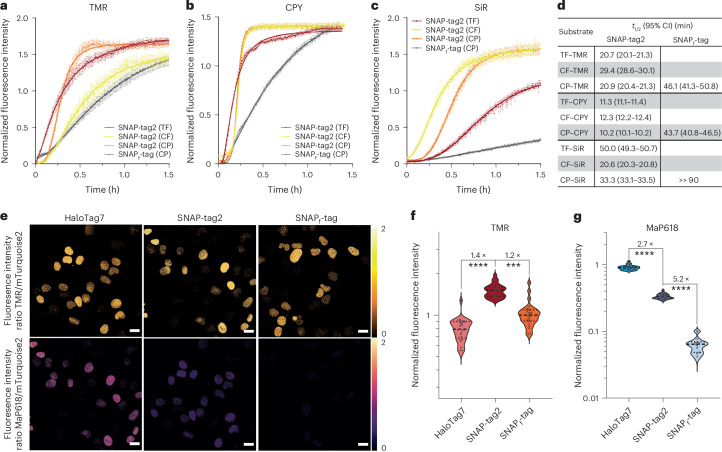
Labeling kinetics and fluorescence brightness for SNAP-tag2 in live cells. Experiments were conducted in live U2OS cells stably coexpressing HaloTag7–SNAP_f_-tag or HaloTag7–SNAP-tag2 together with mTurquoise2 (expression marker) in the nucleus. **a**–**c**, Kinetic traces of SNAP-tag2 and SNAP_f_-tag fluorescence labeling in live cells with TMR (**a**), CPY (**b**) and SiR (**c**) substrates. U2OS cells were labeled with TF–fluorophore, CF–fluorophore and CP–fluorophore substrates (50 nM for TMR and CPY, 100 nM for SiR) and the labeling reaction was followed by confocal fluorescence microscopy. Fluorescence intensity changes of the substrates were normalized to the mTurquoise2 fluorescence over time. Data were fitted to a sigmoidal curve (*n* (TMR) ≥ 66 cells, *n* (CPY) ≥ 105 cells, *n* (SiR) ≥ 27 cells). Data are presented as the mean values ± 95% CI. Representative results from a biological duplicate are shown (replicate in Supplementary Fig. 12). **d**, The *t*_1/2_ of SNAP-tag2 and SNAP_f_-tag calculated from in-cell kinetic measurements described in **a**–**c**. Values represent the mean values ± 95% CI from a biological duplicate. **e**, Comparison of HaloTag7, SNAP-tag2 and SNAP_f_-tag fluorescence brightness in confocal fluorescence microscopy with nonfluorogenic TMR and highly fluorogenic MaP618 substrates. U2OS cells were labeled with SLP substrates CA–TMR, CA-MaP618, TF–TMR and TF–MaP618 (100 nM overnight) and washed before imaging. Ratiometric projections are presented corresponding to fluorescence intensities of label to mTurquoise2 using orange-hot (TMR) and mpl-magma (MaP618) lookup tables. Scale bar, 20 µm. **f**,**g**, Quantitative analysis of single cells shown in **e** represented as violin plots. Numbers represent fold changes between the different SLPs (*n* ≥ 15 cells). Statistical analysis was conducted using a two-tailed unpaired *t*-test with Welch’s correction. ****P < 0.0001 and ***P = 0.0003. Source data

We then tested the performance of SNAP-tag2 and its improved substrates in live-cell imaging applications. We first compared the labeling kinetics of SNAP-tag2 and SNAP_f_-tag with different fluorescent probes (that is, TMR, carbopyronine (CPY) and silicon rhodamine (SiR)) in live U2OS cells. The fluorophores were coupled to CP, CF or TF (Supplementary Fig. 1) to identify the best substrate–fluorophore pair for each fluorophore individually to use in live-cell labeling. We used U2OS cells stably coexpressing nuclear-localized HaloTag7–SNAP-tag2 or HaloTag7–SNAP_f_-tag fusions together with mTurquoise2, which was used for normalization of the protein expression level. Cells were incubated with the respective fluorescent probes and the fluorescence intensity changes were recorded as a function of time. For all tested fluorescent substrates, SNAP-tag2 showed significantly faster labeling than SNAP_f_-tag with CP substrates (Fig. 3a–d and Supplementary Fig. 12). Among the various fluorophores, the differences between SNAP-tag2 and SNAP_f_-tag were most pronounced for the labeling with SiR derivatives (Fig. 3c,d). The half-labeling time (*t*_1/2_) for reaching saturation of the fluorescence signal was around 20 min for SNAP-tag2 labeled with CF–SiR, whereas SNAP_f_-tag showed only relatively weak labeling with CP–SiR under the same experimental conditions, not reaching a plateau within 90 min (Fig. 3c,d). For all CPY substrates, SNAP-tag2 showed around 4-fold faster labeling than SNAP_f_-tag. The *t*_1/2_ values for SNAP-tag2 labeling with different CPY substrates were similar (*t*_1/2_ = 10–12 min), highlighting a small influence of the substrate core for live-cell labeling with this fluorophore. For TMR substrates, the differences between SNAP-tag2 and SNAP_f_-tag were less pronounced (1.6–2.2-fold). This suggests that live-cell labeling with TMR substrates is limited by the entry of the substrate into the cell.

**Extended Data Fig. 3:**
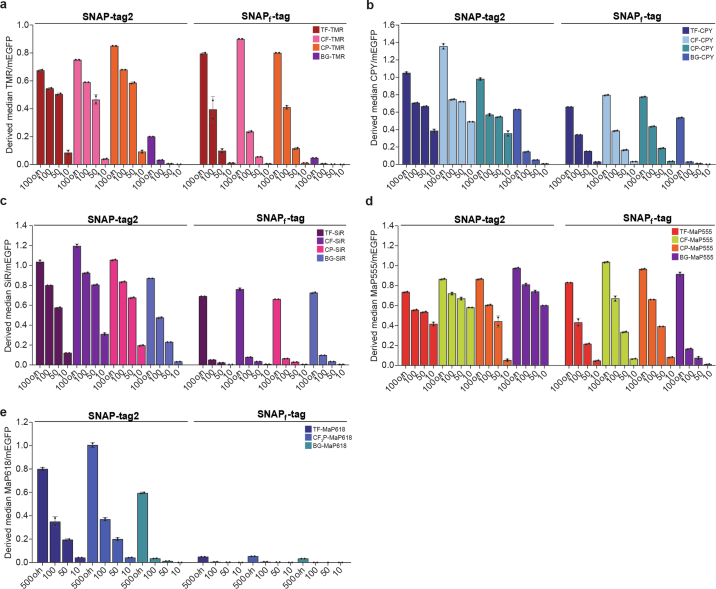
Comparison of SNAP-tag2 und SNAP_f_-tag for labeling with different fluorescent substrates in live cells. U2OS cell stably expressing mEGFP-SNAP-tag2 or mEGFP-SNAP_f_-tag were incubated with different **a** TMR, **b** CPY, **c** SiR, **d** MaP555 or **e** MaP618 substrates at [100 nM] or [500 nM] overnight or at [100 nM, 50 nM and 10 nM] for 1 h. Cells were washed and analyzed by flow cytometry (recorded n = 10000 cells). Analysis was done using FlowJo by gating for single cells and double positive labeling signal (as depicted in Supplementary Fig. 16) and the median of the fluorescent label/mEGFP was derived. Data are represented as median values ± mean s.d. of technical duplicates. SNAP-tag2 showed for most of the substrates a higher labeling ratio. Source data

Similar results were obtained in a flow cytometry-based assay for the labeling of mEGFP–SNAP-tag2 and mEGFP–SNAP_f_-tag fusion proteins in U2OS cells. All tested substrates led to a more efficient labeling of SNAP-tag2 than SNAP_f_-tag, with the most pronounced differences observed for SiR and MaP618 substrates (Extended Data Fig. 3). BG substrates, however, showed reduced labeling in live cells in general (Extended Data Fig. 3).

**Extended Data Fig. 4:**
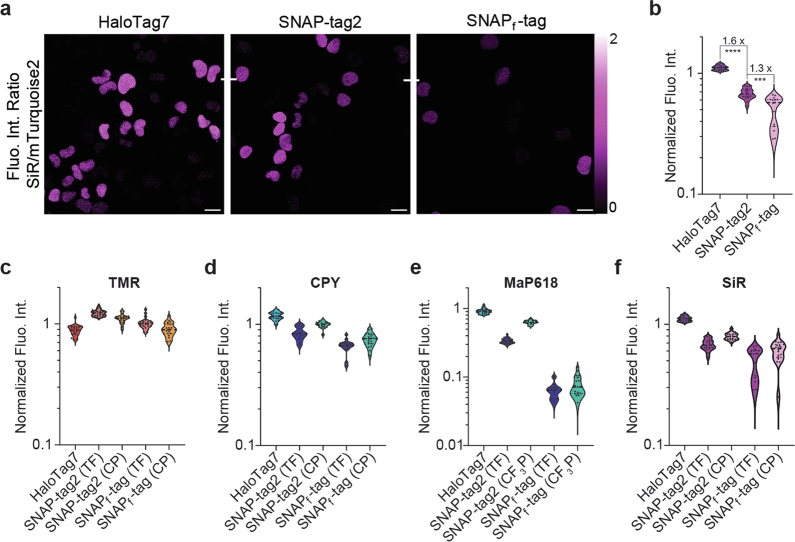
Comparison of HaloTag7, SNAP-tag2 and SNAP_f_-tag performances in confocal fluorescence microscopy. Experiments were conducted in live U2OS cells stably coexpressing HaloTag7-SNAP_f_-tag or HaloTag7-SNAP-tag2 together with mTurquoise2 (expression marker) in the nucleus. **a**, Comparison of HaloTag7, SNAP-tag2 and SNAP_f_-tag performances in confocal fluorescence microscopy with SiR substrates. U2OS cells were labeled with SLP-respective CA-/TF-SiR substrates at [100 nM] overnight and washed afterward. Ratiometric projections are presented corresponding to SiR label/mTurquoise2 on a magenta-hot look-up table. Scale bar: 20 µm. **b**, Violin plots representing the quantitative analysis of single cells shown in a. Numbers represent fold-changes between the different SLPs (n ≥ 15 cells, two-tailed unpaired t-test with Welch’s correction: ****P < 0.0001, ***P = 0.0003). **c-f**, Violin plots representing the quantitative analysis of different fluorescent substrates/mTurquoise2 for **c** TMR, **d** CPY, **e**, MaP618 and **f** SiR (n ≥ 13 cells). Source data

Moreover, we compared the fluorescence brightness of labeled SNAP-tag2 in live cells to that of SNAP_f_-tag and HaloTag7. Previously described U2OS cells stably coexpressing nuclear-localized HaloTag7–SNAP-tag2 or HaloTag7–SNAP_f_-tag fusions together with mTurquoise2 were labeled overnight with CA–TMR, CA-MaP618, TF–TMR or TF–MaP618 to achieve full labeling (Fig. 3e). Quantification of the SLP fluorescence signal normalized to mTurquoise2 expression showed smaller differences among all three SLPs for TMR labeling (Fig. 3f and Extended Data Fig. 4c). Similar results were obtained for SiR substrates (Extended Data Fig. 4a,b,f) and CPY substrates (Extended Data Fig. 4d), for which differences in the fluorescence intensities among all SLPs, including HaloTag7, remained below twofold. For labeling with TF–MaP618, SNAP-tag2 showed a 5.2-fold higher fluorescence intensity than SNAP_f_-tag in cells (Fig. 3g and Extended Data Fig. 4e), which is in line with the measured absorbance difference in vitro (Fig. 2e). However, the brightness of MaP618-labeled SNAP-tag2 remained 2.7-fold dimmer than that of MaP618-labeled HaloTag7. Thus, SNAP-tag2 probably shifts the equilibrium of spirocyclization of very closed rhodamine dyes more toward the zwitterionic form than SNAP_f_-tag but less than HaloTag7.

**Fig. 4:**
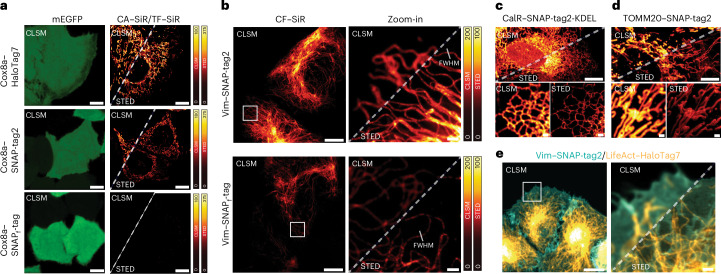
SNAP-tag2 performance in live-cell super-resolution microscopy. **a**, Comparison of HaloTag7, SNAP-tag2 and SNAP_f_-tag performance in CLSM and STED microscopy. HeLa cells stably coexpressing HaloTag7, SNAP-tag2 or SNAP_f_-tag in the mitochondria (Cox8a localization sequence) together with mEGFP (no specific localization) were labeled with CA–SiR or TF–SiR (100 nM) for 1 h and washed afterward. SNAP-tag2 and HaloTag7 show comparable performance in STED imaging, while SNAP_f_-tag shows insufficient signal under the same imaging conditions. This experiment was performed three times with similar results. Scale bars, 10 µm. Lookup tables: green (mEGFP) and red-hot (SiR). **b**,**c**, CLSM and STED images of U2OS cells stably expressing Vim–SNAP-tag2 (**b**) or Vim–SNAP_f_-tag (**c**) labeled with CF–SiR (100 nM) for 1 h. Cells were washed before imaging. White squares in the overview images (left) highlight the area chosen for magnification and STED imaging (right). The experiment was repeated twice with similar results. The FWHM of single intermediate filament fibers highlighted in **b** was determined to be 108 (±23) nm and 146 (±7.0) nm, underlining the higher resolution achieved with SNAP-tag2 compared to SNAP_f_-tag (15 individual filaments from three individual images per condition). Scale bars, 5 µm (overview) and 1 µm (magnification). **c**,**d**, CLSM and STED images of U2OS cells stably expressing SNAP-tag2 (**c**) in the lumen of the endoplasmic reticulum (CalR–KDEL) and (**d**) on the outer mitochondrial membrane (TOMM20) labeled with CF–SiR (100 nM) for 1 h, demonstrating the versatility of using SNAP-tag2 in different cellular compartments. Cells were washed before imaging. Scale bars, 5 µm (overview) and 1 µm (magnification). **e**, Dual-color CLSM and STED images of U2OS cells expressing Vim–SNAP-tag2 and LifeAct–HaloTag7. SNAP-tag2 was labeled with CF–SiR (100 nM) and HaloTag7 was labeled with CA–MaP618 (100 nM) for 1 h; cells were washed afterward. Scale bars, 5 µm (overview) and 1 µm (magnification). Lookup tables: cyan (SNAP-tag2–SiR) and orange-hot (HaloTag7–MaP618).

**Extended Data Fig. 5:**
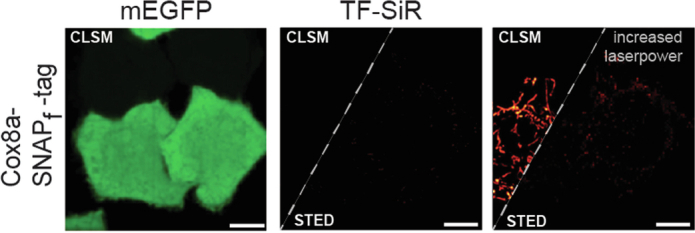
SNAP_f_-tag performance in CLSM and STED microscopy. HeLa cells stably co-expressing SNAP_f_-tag in the mitochondria (Cox8a localization sequence) together with mEGFP (no specific localization) were labeled with TF-SiR (100 nM) for 1 h and washed afterward. SNAP_f_-tag showed insufficient signal under the same imaging conditions (0.5 % laser power) used for SNAP-tag2 and HaloTag7 and required the use of an increase laser power (5 %) to see a weak labeling signal. Scale bars: 10 µm. LUTs: green (mEGFP), red-hot (SiR).

We further tested the performance of SNAP-tag2 in super-resolution stimulated emission–depletion (STED)^29^ microscopy. HeLa cells stably coexpressing HaloTag7, SNAP-tag2 or SNAP_f_-tag fused to the Cox8a presequence (mitochondrial matrix localization) and mEGFP as an expression marker (no specific localization) were labeled with their respective CA–SiR or TF–SiR substrates (Fig. 4a). SNAP-tag2 showed much stronger fluorescence labeling inside mitochondria, both in confocal laser scanning microscopy (CLSM) and STED microscopy than observed for SNAP_f_-tag under the same experimental conditions, even though a cell with stronger mEGFP fluorescence (≙ higher expression level) was chosen for SNAP_f_-tag. Detection of the dim SNAP_f_-tag labeling required a significant increase in laser power (Extended Data Fig. 5). The observed fluorescence signal for SNAP-tag2 was comparable to the signal obtained for the corresponding HaloTag7 labeling at similar expression levels. Similar differences in fluorescence intensities were observed for SNAP-tag2 and SNAP_f_-tag labeling of intermediate filaments (vimentin (Vim) fusion) (Fig. 4b). Because of the increased photon count, SNAP-tag2 showed improved STED resolution, as highlighted by the reduced full-width at half-maximum (FWHM) of Vim filaments (108 ± 23 nm) compared to SNAP_f_-tag (146 ± 7.0 nm). We also tested the STED performance of SNAP-tag2 with other fusion proteins such as CalR–KDEL (endoplasmic reticulum lumen; Fig. 4c) and TOMM20 (outer mitochondrial membrane; Fig. 4d) and observed bright and specific labeling of SNAP-tag2 with CF–SiR. Furthermore, we performed dual-color live-cell STED imaging in U2OS cells expressing Vim–SNAP-tag2 and LifeAct–HaloTag7 (F-actin) using CF–SiR and CA–MaP618, respectively (Fig. 4e). These results demonstrate the superiority of SNAP-tag2 over previous SNAP-tag versions in live-cell imaging experiments.

**Fig. 5:**
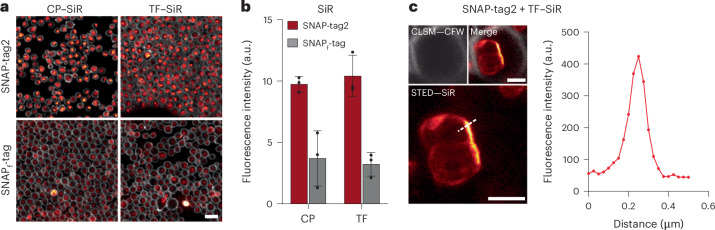
Comparison of SNAP-tag2 and SNAP_f_-tag labeling of yeast peroxisomes. **a**, CLSM images of *H.* *polymorpha* yeast cells expressing Pex3–SNAP-tag2 or Pex3–SNAP_f_-tag fusion proteins labeled with different SiR substrates. Yeast cells were labeled with CP–SiR or TF–SiR (250 nM) for 18 h and the cell wall was stained with Calcofluor white (CFW; 25 µg ml^−1^) for 15 min. Cells were washed before imaging. Scale bar, 10 µm. **b**, Bar plot representing the quantitative analysis of SNAP-tag2 and SNAP_f_-tag labeling with SiR substrates in yeast. Experiments were conducted in biological triplicates (*n* = 125 cells for each replicate) and the mean fluorescence intensity of SiR substrates was calculated (Supplementary Fig. 14). Error bars represent the s.d. SNAP-tag2 greatly outperforms SNAP_f_-tag in labeling of live yeast peroxisomes. **c**, Bottom, STED image of Pex3–SNAP-tag2 labeled with TF–SiR. Top, CLSM image of CFW-stained cell wall and merge of both channels. Scale bar, 1 µm. Right, line profile of labeled peroxisomes in STED imaging (highlighted as dashed line in the image). SNAP-tag2 with TF–SiR is suitable to perform live-cell STED microscopy in yeast. Source data

**Extended Data Fig. 6:**
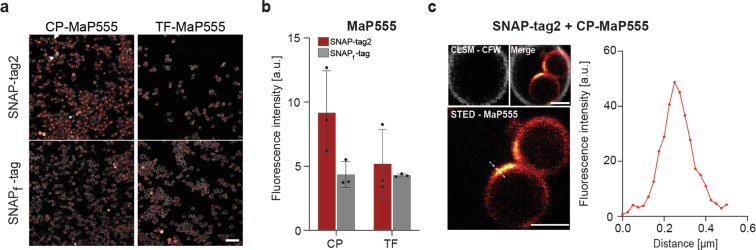
SNAP-tag2 and SNAP_f_-tag labeling of live yeast peroxisomes. **a**, CLSM images of *H. polymorpha* yeast cells expressing Pex3-SNAP-tag2 or Pex3-SNAP_f_-tag fusion proteins labeled with different MaP555 substrates. Yeast cells were labeled with CP- or TF-MaP555 (250 nM) for 18 h and the cell wall was stained with Calcofluor White (CFW; 25 µg/mL) for 15 min. Cells were washed prior to imaging. Scale bar: 10 µm. **b**, Bar plot representing the quantitative analysis of SNAP-tag2 and SNAP_f_-tag labeling with MaP555 substrates in yeast. Experiments were conducted in biological triplicates (n = 125 cells for each replicate) and the mean fluorescence intensity of MaP555 substrates was calculated (Supplementary Fig. 14). Error bars represent the s.d. Combination of SNAP-tag2 with CP-MaP555 show the best results for labeling of yeast peroxisomes. **c**, Bottom, STED image of Pex3-SNAP-tag2 labeled with CP-MaP555. Top, CLSM image of CFW stained cell wall and merge of both channels. LUTs: red-hot (MaP555) and gray (CFW). Scale bar: 1 µm. Right, line profile of labeled peroxisomes in STED (highlighted as white dashed line in the image). SNAP-tag2 with CP-MaP555 is well suited to perform live cell STED in yeast. Source data

We then investigated whether the improved SNAP-tag2 system translates to more efficient fluorescence labeling in yeast. Chemical labeling in yeast is challenging, presumably because of the presence of its cell wall, an additional permeability barrier and the expression of various multidrug efflux pumps^30^. The peroxisomal membrane protein Pex3 has a key role in peroxisomal biogenesis, inheritance and degradation in yeast^31^. To study the precise localization, dynamics and distribution of Pex3 and its binding partners, live-cell STED nanoscopy is a suitable technique because it enables the quantification of the exact number and size of peroxisomes in live yeast^32^. Thus, we expressed either Pex3–SNAP-tag2 or Pex3–SNAP_f_-tag in the methylotrophic yeast species *Hansenula polymorpha* and tested different SiR substrates for labeling of peroxisomes in live cells (Fig. 5a). *H.* *polymorpha* expressing Pex3–SNAP-tag2 showed homogenous and highly efficient labeling of peroxisomes, whereas the fluorescence signal was much weaker for yeast cells expressing Pex3–SNAP_f_-tag (Fig. 5a,b). The correct localization of Pex3–SNAP-tag2 was furthermore confirmed through STED microscopy (Fig. 5c and Supplementary Fig. 13a,b). Similar results were obtained for labeling with MaP555 (ref. ^9^) substrates, for which SNAP-tag2 worked best in combination with CP–MaP555 (Extended Data Fig. 6 and Supplementary Fig. 13c,d). These results demonstrate that SNAP-tag2 enables more efficient labeling in *H.* *polymorpha* yeast than its predecessor.

## Discussion

In this work we used a combination of substrate optimization and protein engineering to develop SNAP-tag2, an improved version of the SNAP-tag labeling system. SNAP-tag2 reacts faster with an improved set of pyrimidine-based substrates. An increased labeling speed is observed for a large variety of probes, including rhodamines as well as different reactive biorthogonal groups. The *k*_app_ for the reaction of SNAP-tag2 with the substrate TF–TMR in vitro approaches 10^7^ M^−1^ s^−1^, which is ~100-fold faster than the labeling reaction of SNAP-tag with CP–TMR and is close to that of HaloTag7 with CA–TMR. SNAP-tag2 remained a very stable protein with a melting temperature of ~65 °C, which might facilitate further engineering attempts such as circular permutations and the generation of split versions of SNAP-tag2. The creation of such cpSNAP-tag2 and split-SNAP-tag2 systems could enable the development of biosensors and recorders for studying biological activities in cells and/or in vivo^33^. Its size of 172 aa is slightly below that of its predecessor and substantially below that of HaloTag7 with 297 aa. SNAP-tag2 displaces the spirocyclization equilibrium of bound rhodamines more toward the open form compared to SNAP_f_-tag, a property for which we directly screened in the directed evolution experiments. We assume that a possible interaction of SNAP-tag2 with the zwitterionic, fluorescent configuration of rhodamines results in higher brightness of SNAP-tag2 when labeled with the very fluorogenic dye MaP618. Nevertheless, SNAP-tag2 displaces the spirocyclization equilibrium to a lower degree than HaloTag7. Since very closed rhodamine derivatives tend to show reduced labeling performance in cellular experiments^15^ and in vivo^34^, we believe that this is not a major limitation of SNAP-tag2 relative to HaloTag7. Owing to the lack of structural information on labeled SNAP-tag2, the role of individual amino acid substitutions in tuning the interactions of SNAP-tag2 with rhodamines remains unclear.

Most importantly, the increased reactivity of SNAP-tag2 toward its improved substrates translates into a more efficient fluorescence labeling in live cells. This observation was most pronounced for fluorescence labeling with the far-red SiR substrates, which enabled efficient fluorescence labeling at substrate concentrations that only resulted in weak labeling of SNAP_f_-tag. The relatively weak SiR labeling observed for SNAP_f_-tag is in line with a previous report on differences of SNAP-tag and HaloTag7 labeling with SiR substrates^22^. In live-cell imaging, the different fluorophores showed some preferences among the different SNAP-tag substrates. For SiR, CF–SiR showed faster labeling in U2OS cells than TF–SiR and CP–SiR, even though all reached the same brightness after longer incubation periods. The labeling of SNAP-tag2 in live cells with TMR substrates and CPY substrates showed less dependency on the nature of the substrate (TF, CF and CP). The *k*_app_ values of CPY substrates for labeling SNAP-tag2 in vitro are all >10^6^–10^7^ M^−1^ s^−1^ with TF–CPY being ~2-fold faster in comparison to CF–CPY and CP–CPY. The fact that the *t*_1/2_ values of all CPY substrates for labeling in cells are rather similar suggests that the lower reactivity of CP–CPY and CF–CPY is offset by higher intracellular concentration of these substrates. This might be because of higher permeability and/or reduced binding to other cellular components. Thus, for the preparation of any new probes for live-cell applications with SNAP-tag2 or for new applications of the here-described probes, we recommend to first test the CF-based probes. The greater performance of SNAP-tag2 for fluorescence labeling in live cells also extends to super-resolution microscopy imaging techniques such as STED microscopy and to other cell types known to be refractory to chemical labeling such as yeast cells. As SNAP-tag2 together with its improved substrates is superior to the previously used SNAP-tag versions in every application we tested, we expect that it will also excel in in vivo applications. Lastly, implementation of the superior SNAP-tag2 system to already existing SNAP-tag-based biosensors such as Snifits^35^ should enhance their performance.

In summary, the improvements introduced in SNAP-tag2 are expected to advance the utility of this already widely adopted tool for live-cell imaging and other applications in life sciences.

## Methods

### Chemical synthesis and general methods

Detailed procedures for the synthesis and chemical characterization of all compounds are given in the Supplementary Methods. Buffer, media and reagent compositions can be found in Supplementary Table 13.

### Computed chemical properties of SNAP-tag substrates

Molecular structures were prepared using the Schrödinger Maestro 12.3 interface to ensure suitability for QikProp calculations. The preparation included adding hydrogens, assigning bond orders and generating three-dimensional geometries. QikProp calculations were conducted using the Schrödinger suite. For each structure, key pharmacokinetic and physicochemical properties were calculated using default settings and according to the official protocol (https://www.schrodinger.com/platform/products/qikprop/). Calculated parameters were exported and are shown in Supplementary Table 2.

### Plasmids for bacterial and mammalian protein expression

A pET51b(+) vector (Novagen) was used for protein production in *Escherichia coli*. Proteins were N-terminally Strep-tagged and C-terminally 10xHis-tagged for affinity purification. pcDNA5/FRT or pcDNA5/FRT/TO vectors (Thermo Fisher Scientific) were used for protein expression in mammalian cells by transient transfection or for stable cell line generation. Cloning was performed with Gibson assembly^37^ or point mutations were introduced using the Q5-site-directed mutagenesis kit (New England Biolabs) according to the manufacturer protocol. Plasmid sequences were verified by Sanger sequencing (Eurofins Genomics or Microsynth) and plasmids were stored at −20 °C.

### Recombinant protein production and purification

pET51b(+) plasmids were transformed in *E.* *coli* BL21(DE3)-pLysS (Novagen) cells. Luria–Bertani (LB) ampicillin expression cultures were grown at 37 °C to an optical density at 600 nm (OD_600_) of 0.6–0.8 and protein expression was subsequently induced at 16 °C by addition of IPTG (0.5 mM). After overnight expression, cells were harvested by centrifugation (4,000*g*, 4 °C, 15 min), resuspended in His-tag extraction buffer (20–30 ml) and lysed by sonication (50% duty, 70% power, 7 min) on wet ice. All proteins were purified by immobilized metal ion affinity chromatography using either a gravity column or an ÄktaPure fast protein liquid chromatography (FPLC) instrument (Cytiva) equipped with a HisTrap FF crude column (Cytiva). Buffer was exchanged to activity buffer (50 mM HEPES and 50 mM NaCl, pH 7.3) either using Zeba spin desalting columns (7-kDa molecular weight cutoff (MWCO), 0.5 ml; Thermo Fisher Scientific) or HiPrep 26/10 desalting columns (Cytiva) on an FPLC system. Purified proteins were concentrated using Amicon Ultra centrifugal filters (10-kDa MWCO). The purity and correct size of the proteins were assessed by SDS–PAGE and high-resolution mass spectrometry (HRMS). Purified proteins were aliquoted, flash-frozen in liquid nitrogen and stored at −80 °C.

### Plasmids for YSD

For protein expression on the yeast surface, either a pCTcon2 vector (Addgene, 73152) with SNAP-tag proteins fused in between Aga2p–HA-tag (N-terminal) and MYC-tag (C-terminal) or a pJYDNg vector (Addgene, 162452) with a C-terminal fusion to Aga2p–HA-tag–MYC-tag–eUnaG2 was used. Epitope HA-tags and MYC-tags were used for immunostaining and the fluorescent reporter protein eUnaG2 was labeled with bilirubin for monitoring the protein expression level.

### DNA library preparation for YSD

Libraries were created either using pCTcon2-SNAP-tag1.1 (SNAP-E30R-S135R-L153G) through one-pot saturation mutagenesis^38^ or using pJYDNg-SNAP-tag1.5 (SNAP-E30R-I32Y-L34V-K36N-^50^GHPEPQ-S135R-L153G-G161P) combining site-directed saturation mutagenesis with assembly PCR^39^.

Library generation was initially performed by obtaining circular library plasmids from *E.* *coli*, which were then used for transformation into EBY100 yeast cells for YSD. For libraries generated through one-pot saturation mutagenesis, a mixture of degenerate primers was used, in which one or four NNKs covered the target regions for randomization (amino acids H29–K36 and 156–161; Supplementary Tables 15–17). Library DNA was transformed into *E.* *coli* cells and cells were recovered in 2 ml of SOC medium (New England Biolabs) for 1 h at 37 °C. Serial dilutions were spread on selective LB agar plates containing kanamycin (50 µg ml^−1^) to determine the library size. Plates were incubated at 37 °C overnight and isolated plasmids from ten single-picked colonies were sequenced to control the library diversity. Cells transformed with the libraries were grown in 3 ml of LB kanamycin medium at 37 °C overnight and plasmids were extracted using a QIAprep Spin Miniprep kit (Qiagen).

Libraries were later generated as linear DNA fragments used for homologous recombination (HR) in yeast. Libraries on the active site loop of pJYDNg-SNAP-tag1.5 were generated using degenerated primers with three NNKs at different positions throughout the targeted region (156-GGYEGP-161). The N-terminal and C-terminal parts of SNAP-tag1.5 were PCR-amplified and used for assembly PCR in a mixture of primers carrying randomized positions surrounded by overlapping regions with the N-terminal and C-terminal fragments (Supplementary Table 18). This assembly PCR enabled the reconstitution of full-length SNAP-tag1.5 library genes, whose extremities overlapped with the plasmid by 50–80 bp to enable HR in yeast. The protocol for assembly PCR was adjusted from Routh et al.^39^. Library inserts were purified using a QIAquick PCR purification kit (Qiagen).

An sDMSL designed on the SNAP-tag1.5 scaffold was purchased from Twist Bioscience. The library consisted of single-point variants, having each amino acid along the SNAP-tag1.5 sequence substituted to all other 19 amino acids, excluding cysteines and including deletions. The sDMSL was designed with a 50-bp overlap of the flanking regions to the pJYDNg expression vector for direct HR into yeast cells.

### Electrocompetent yeast cell preparation for YSD

Single colonies of *Saccharomyces cerevisiae* strain EBY100 (American Type Culture Collection) spread on a YPD agar plate were used for inoculation of YPD liquid cultures. YPD expression cultures were grown at 250 rpm and 30 °C until an OD_600_ of 1.3–1.5. Afterward, Tris–DTT buffer (800 µl) and Tris–lithium acetate buffer (2 ml) were added and the culture was incubated at 250 rpm and 30 °C for 15 min. Cells were harvested by centrifugation (2,500*g*, 4 °C, 3 min) and washed with ice-cold electroporation buffer (25 ml), followed by cell harvesting (2,500*g*, 4 °C, 3 min). Cells were resuspended in electroporation buffer to a total volume of 300 µl. Cells were aliquoted (50 µl) and directly used for transformation by electroporation.

### Yeast cell transformation and HR for YSD

For circular plasmid DNA transformation, DNA (1–3 µg) was diluted in double-distilled (dd)H_2_O (<10 µl) and kept on ice. For HR, amplified vector (1 µg) and insert DNA (2–3 µg) were mixed to a final volume of 100 µl. The DNA was concentrated using alcohol precipitation (100 µl of isopropanol 100 µl, 10 µl of 3 M sodium acetate and 0.5 µg µl^−1^ glycogen (Thermo Fisher Scientific), incubated at −20 °C overnight). The mixture was centrifuged (10,000*g*, 20 min) and the DNA pellet was washed with 70% ethanol (200 µl) followed by centrifugation (10,000*g*, 20 min). The supernatant was removed and the pellet was air-dried. Electrocompetent EBY100 yeast cells (50 µl) were used to resuspend the DNA and transformation was conducted by electroporation. Electroporation cuvettes (GenePulser cuvette, 0.2-cm electrode gap; Bio-Rad Laboratories) were prechilled on ice. The cell–DNA mix was transferred to the prechilled electroporation cuvette and electroporation was conducted using a Bio-Rad GenePulser Xcell device (0.54 kV, 25 µF, infinite resistance with exponential decay). After electroporation, prewarmed YPDS medium (1 ml) was immediately added to the cuvette and the cell suspension was transferred to culture tubes (15 ml). The cuvette was washed with 1 ml of YPDS medium and added to the culture. The culture was incubated at 250 rpm and 30 °C for 1 h. Yeast cells were collected by centrifugation (2,500*g*, 5 min) and resuspended in SDCAA selective medium (1 ml). Serial dilutions were plated on SDCAA agar plates to determine the transformation efficiency. Plates were incubated at 30 °C for 2–3 days. The remaining cell suspension was used to inoculate SDCAA culture (100 ml) and grown at 30 °C for 2 days. The culture was directly used for YSD screening and aliquots were frozen in a 1:1 mixture of culture and 50% glycerol in Tris buffer (pH 8).

### Protein expression on the yeast surface

SDCAA medium (5 ml final volume) was inoculated either using a single colony from a SDCAA plate or by addition of fully grown yeast liquid culture (0.5 ml) after transformation. The culture was incubated at 250 rpm and 30 °C overnight. This preculture (0.5 ml) was used to inoculate 4.5 ml of SGCAA medium for protein expression on the yeast surface. Protein expression was conducted for at least 20 h at 250 rpm and 30 °C.

### Protein labeling on yeast surface for FACS

For fluorescence labeling, 10^7^ cells were obtained by centrifugation (14,000*g*, 1 min). The concentration of the yeast culture expressing the protein(s) of interest on the surface was determined by accounting that an OD_600_ = 1 corresponds to about 10^7^ cells per ml (ref. ^40^). For antibody-based expression staining of yeast cells transformed with pCTcon2 plasmids, yeast cells were resuspended in 1:10-diluted primary mouse anti-MYC antibody (OP10, EMD Millipore) in PBS (50 μl) and incubated on a rotating wheel at 4 °C for 1 h. The cells were pelleted (14,000*g*, 1 min) and washed twice with PBS (125 μl) with centrifugation in between (14,000*g*, 1 min). The cell pellet was resuspended in 1:50-diluted secondary goat anti-mouse–Alexa647 antibody (A-21236, Invitrogen, Thermo Fisher Scientific) in PBS (50 μl) and incubated on a rotating wheel at 4 °C for 1 h. The cells were washed twice with PBS (125 μl) before labeling with SNAP-tag substrates.

For protein libraries encoded by the pJYDNg expression vector, expression control was monitored over labeling of eUnaG2 with bilirubin. Yeast cells were resuspended in PBS (50 μl) containing bilirubin (10 μM) and BSA (1 mg ml^−1^), gently vortexed and incubated on ice for 10 min. The cells were pelleted and washed twice with PBS (150 μl) interspaced by centrifugation steps (14,000*g*, 1 min) before SNAP-tag labeling. Cells expressing SNAP-tag variants were labeled in PBS (50 μl) using different substrates (CF_3_P–TMR, CF_3_P–MaP618, TF–TMR or TF–MaP618) with varying concentrations (10–500 nM) and incubation times (10–60 min) to adjust the screening stringency. Labeling was performed at room temperature on a rotating wheel. Cells were washed with PBS (125 μl), resuspended in 1 ml of PBS and filtered through 5-ml round-bottom polystyrene test tubes with cell strainer snap caps (352235, Falcon) for FACS.

### Protein library screening using FACS

Labeled yeast libraries were analyzed and subsequently sorted on a BD FACSMelody cell sorter (BD Biosciences) using the appropriate filter settings for each fluorophore (Supplementary Table 7). For yeast cell sorting, a 100-μm sorting nozzle and a 1.5 neutral density filter was used. Photomultiplier tube (PMT) detector voltages were optimized on the basis of single-labeled and double-labeled samples and negative control samples. The range for positive signal was set to be approximately 10^3^–10^4^, while negative and background staining was set to ≤10^2^. Gating strategies are depicted in Supplementary Fig. 15. Cells were gated for live and single-cell events on the basis of their size in the forward scatter (FCS) and side scatter (SSC). The single-cell population was then gated for the double-positive labeling signal and the top 0.5–1% of double-labeled cells (10^4^ cells) were sorted in bulk. After sorting, yeast cells were grown in SDCAA medium (5 ml) supplement with penicillin–streptomycin (Gibco; 5,000 U per ml, 1:100 dilution) for 2 days. Plasmids were isolated from fully grown cultures (1 ml) using a Zymoprep yeast plasmid miniprep II kit (Zymo Research) according to the manufacturer’s protocol and the remaining culture was propagated to start the next screening round.

### Computational redesign of the unstructured loop in SNAP-tag

To redesign the unstructured loop (residues 37–54), the TMR-labeled SNAP-tag structure (PDB 6Y8P)^16^ was used as input. The covalent benzyl-TMR ligand was parameterized and a Rosetta constraint file was set up to fix the covalent bond between the ligand the protein analogous to published procedures^33^. Point substitutions E30R, I32Y, L34V, K36N, S135R, L153G and G161P were modeled and the structure was minimized using a RosettaScripts^41^ protocol. The top-scoring structure was used as input for the loop design using the RosettaRemodel^27^ application. Blueprint files for 7-aa, 8-aa and 9-aa loops were set up, allowing any amino acids except cysteine in the designed regions. For energy minimization, the Rosetta FastRelax method was applied using the ‘ref2015’ Rosetta score function and the limited-memory Broyden–Fletcher–Goldfarb–Shanno algorithm with Armijo inexact line search conditions (‘lbfgs_armijo_nonmonotone’). Example scripts can be found in the Supplementary Information. The top five scoring sequences for each loop length were tested experimentally.

### Next-generation sequencing

Randomized regions from isolated plasmids were amplified with next-generation sequencing (NGS) primers (Supplementary Table 19) using standard PCR conditions and purified (Qiagen PCR purification kit). NGS was performed using adaptor ligation Illumina sequencing at Eurofins Genomics. The NGS package for each sample included 10 million total reads comprising of 2× 150-bp paired-end reads (5 million read pairs). NGS amplicon sizes varied from 150 to 300 bp and were pooled in equivalent quantities. The NGS primers’ unique adaptor sequences enabled later reattribution of sequences to sorted libraries. NGS samples comprised 2 μg of DNA in 100 μl of ddH_2_O.

### Analysis of NGS data

Illumina sequencing results were first split into separate files on the basis of their experiment-specific and selection-round-specific sequence barcodes using ‘je demultiplex’ (ref. ^42^). Forward and reverse reads were combined into a single file for each barcode. Primer and adaptor sequences were trimmed to match the translation frame of the template sequence using cutadapt^43^. Trimmed reads were then aligned to the template sequence with bowtie2 (ref. ^44^). The resulting SAM file was converted to a BAM file using SAMtools^45^. The alignments were then analyzed with a custom R script to calculate amino acid frequencies for randomized positions (for saturation mutagenesis libraries) or point mutation frequencies (for sDMSL). Reads containing deletions, insertions relative to the template sequence or ambiguous bases were removed. The remaining reads were translated into amino acid sequences. For saturation mutagenesis libraries, amino acid frequencies at the positions of interest were calculated by dividing the number of reads featuring a specific amino acid at a particular position by the total number of reads. For DMSLs, mutation frequencies were calculated by dividing the number of reads featuring a specific mutation by the number of reads that cover the corresponding position. The script used for NGS data analysis can be found in the Supplementary Information.

### Protein thermal stability analysis

Thermal stabilities of final protein variants were measured on a Prometheus NT48 nanoscale differential scanning fluorimeter (NanoDSF). Protein samples were prepared in activity buffer (0.8 mg ml^−1^) and changes in tryptophan fluorescence at 330 nm were followed over a temperature range of 20–95 °C with a temperature increase of 1 °C min^−1^. Measurements were performed in technical triplicates. The inflection point of the first derivative corresponds to the proteins’ melting temperature.

### In vitro labeling kinetics measured using a microplate reader fluorescence polarization assay

Labeling kinetics were measured in black nonbinding flat-bottom 96-well plates (200-μl final reaction volume) or in black nonbinding low-volume 384-well plates (20-μl final reaction volume) (Corning) on a microplate reader (Spark 20M, Tecan) by recording fluorescence polarization (FP) traces at 37 °C over time. All measurements were performed in technical triplicates in FP buffer. Labeling reactions were started by either addition of substrate to the protein using a multichannel pipette or using the injector module of the plate reader for fast reaction conditions. Filter settings were chosen according to the fluorophore substrate (Supplementary Table 8). The *G* factors were calculated using a buffer only (blank) and free fluorophore substrate (reference) control. The optical gain, an amplification factor for the photomultiplier tube, was adjusted to 50%. Baselines were determined by recording FP kinetic traces of the free fluorophore substrates.

For screening purposes, labeling kinetics were recorded using 50 nM protein and 20 nM fluorophore substrate. For fast labeling kinetics, FP kinetics were conducted with 10 nM protein and 2 nM substrate in 384-well plates. A one-phase association (Eq. (2)) was fitted to the data using GraphPad Prism and *k*_app_ was calculated using Eq. (1).

1 $$Y={Y}_{0}+({Y}_{\max }-{Y}_{0})\times\left(1-{e}^{(-{kx})}\right)$$

where *Y* is the FP (mP), *Y*_max_ is the FP plateau (mP), *Y*_0_ is the *y*-intercept (mP), *k* is the labeling rate constant (s^−1^) and *x* is time (s).

2 $${k}_{\rm{app}}=\frac{k}{[\rm{protein}]}$$

For more accurate determination of *k*_app_, a global fit approach to kinetic models was used as published in Wilhelm et al.^16^. In brief, kinetics were measured at fixed fluorophore substrate concentration (20 nM) and various protein concentrations (0 and 4.60–900 nM). Fast kinetics were measured at fluorophore substrate concentrations of 2 nM and protein concentrations of 0 and 0.78–50 nM. Kinetic data were preprocessed using a custom R script^46,47^; subsequently, kinetic model 1 (Eq. (3)), described by the differential equations (Eqs. (4)–(6)), was fitted to the data using DynaFit^48^. The FP baseline value and protein concentration were fixed parameters, while the fluorophore substrate concentration was adjustable to account for potential quantification errors. Including limiting protein concentration into the experimental conditions enabled accurate concentration fitting by impacting the final FP value (decreasing plateau). The s.d. and confidence interval (CI) were obtained using a Monte Carlo simulation^49^ with standard settings (*n* = 1,000, 5% worst fits excluded). Exemplified DynaFit scripts can be found in the Supplementary Information. Representative graphics were generated using a custom R script (Supplementary Fig. 2).

3 $$P+S\mathop{\longrightarrow}\limits^{{k}_{\rm{app}}}{PS}$$

where *P* is the SNAP-tag2 protein, *S* is the fluorophore substrate and *PS* is the fluorescently labeled SNAP-tag2.

4 $$\frac{{\rm{d}}[P]}{{\rm{d}}t}=-{k}_{\rm{app}}[P][S]$$

5 $$\frac{{\rm{d}}[S]}{{\rm{d}}t}=-{k}_{\rm{app}}[P][S]$$

6 $$\frac{{\rm{d}}[{PS}]}{{{\rm{d}}t}}={k}_{\rm{app}}[P][S]$$

Labeling kinetics of nonfluorescent substrates were determined in a competition assay as described in Wilhelm et al.^16^. In brief, competition kinetics were measured by mixing varying concentrations of nonfluorescent substrates (0 and 0.1– 1 µM) and BG–Alexa488 (100 nM) followed by addition of protein (200 nM) using an electronic 96-channel pipettor (Integra Bioscience). FP changes were recorded over time. Kinetic data were preprocessed using a custom R script^46,47^; subsequently, a competition kinetic model (Eq. (7)), described by the differential equations (Eqs. (8)–(12)), was fitted to the data using DynaFit^48^. The s.d. and CI were obtained using a Monte Carlo simulation^49^ with standard settings (*n* = 1,000, 5% worst fits excluded). Exemplified DynaFit scripts can be found in the Supplementary Information. Representative graphics were generated using a custom R script (Supplementary Fig. 10).

7 $$\begin{array}{c}P+S\mathop{\longrightarrow}\limits^{{k(S)}_{\rm{app}}}{PS}\\ P+I\mathop{\longrightarrow }\limits^{{k(I)}_{\rm{app}}}{PI}\end{array}$$

where *P* is the SNAP-tag2 protein, *S* is the nonfluorescent substrate, *PS* is the nonfluorescently labeled SNAP-tag2 protein, *I* is the fluorescent competitor substrate and *PI* is the fluorescently labeled SNAP-tag2 protein.

8 $$\frac{{\rm{d}}[P]}{{{\rm{d}}t}}=-{k\left(S\right)}_{\rm{app}}\left[P\right]\left[S\right]-{k\left(I\,\right)}_{\rm{app}}\left[P\right]\left[I\,\right]$$

9 $$\frac{{\rm{d}}[S]}{{{\rm{d}}t}}=-{k(S)}_{\rm{app}}[P][S]$$

10 $$\frac{{\rm{d}}[I\,]}{{\rm{d}}t}=-{k(I\,)}_{\rm{app}}[P][I\,]$$

11 $$\frac{{\rm{d}}[{PS}]}{{{\rm{d}}t}}={k(S)}_{\rm{app}}[{PS}]$$

12 $$\frac{{\rm{d}}[{PI}\,]}{{{\rm{d}}t}}={k(I\,)}_{\rm{app}}[{PI}\,]$$

### In vitro labeling kinetics of SNAP-tag2 using a stopped-flow device

Labeling kinetics of SNAP-tag2 with TF–TMR, CF–TMR, CP–TMR, TF–CPY, CF–CPY and CP–CPY substrates were measured by recording fluorescence anisotropy changes over time using a BioLogic SFM-400 stopped-flow instrument (BioLogic Science Instruments) in a single-mixing configuration at 37 °C as described in Wilhelm et al.^16^. The final substrate concentration was fixed to 0.5 or 1 μM and the protein concentration was varied from 0.25 to 3.13 μM in activity buffer (50 mM HEPES and 50 mM NaCl, pH 7.3) supplemented with 1 mM DTT and 0.1 mg ml^−1^ BSA. The anisotropy of the free substrate was measured to obtain a baseline. The sampling time was set to 1 ms for a total recording time of 2 s or varied from 200 µs for 0.2 s followed by 2 ms to a total duration of 3 s. For each condition, 15 technical replicates were recorded. Kinetic data were preprocessed using a custom R script^46,47^, in which replicate values were averaged, pretrigger timer points were removed and times were adjusted to start at zero. Kinetic model 2 (Eq. (13)), described by differential equations (Eqs. (14)–(17)) was fitted to the data using DynaFit^48^ as described in Wilhelm et al.^16^. The delay time, anisotropy baseline value and protein concentration were fixed parameters, while the fluorophore substrate concentration was adjustable to account for potential quantification errors. Including limiting protein concentration into the experimental conditions enabled accurate concentration fitting by impacting the final anisotropy value (decreasing plateau). The s.d. and CI were obtained using a Monte Carlo simulation^49^ with standard settings (*n* = 1,000, 5% worst fits excluded). Exemplified DynaFit scripts can be found in the Supplementary Information. Representative graphics were generated using a custom-made R script (Supplementary Figs. 6–9 and 11).

13 $$\begin{array}{c}\quad{k}_{1}\\P+S\leftrightarrow {{PS}}^{* }\\\quad{k}_{-1}\\{{PS}}^{* }\mathop{\to }\limits^{{k}_{2}}{PS}\end{array}$$

where *P* is the SNAP-tag2 protein, *S* is the fluorophore substrate, PS* is the substrate-bound protein complex and *PS* is the fluorescently labeled SNAP-tag2.

14 $$\frac{{\rm{d}}[P]}{{{\rm{d}}t}}=-{k}_{1}[P][S]+{k}_{-1}\left[{{PS}}^{\ast}\right]$$

15 $$\frac{{\rm{d}}[S]}{{{\rm{d}}t}}=-{k}_{1}[P][S]+{k}_{-1}\left[{{PS}}^{\ast}\right]$$

16 $$\frac{{\rm{d}}[{{PS}}^{\ast}]}{{{\rm{d}}t}}={k}_{1}[P][S]-{k}_{-1}\left[{{PS}}^{\ast}\right]-{k}_{2}\left[{{PS}}^{\ast}\right]$$

17 $$\frac{{\rm{d}}[{PS}]}{{{\rm{d}}t}}={k}_{2}\left[{{PS}}^{\ast}\right]$$

The individual kinetic parameters for the dissociation constant (*K*_d_) defining the protein affinity toward the substrate and the *k*_app_ defining the overall labeling speed were derived from Eqs. (18) and (19), respectively.

18 $${K}_{{\rm{d}}}=\frac{{k}_{-1}}{{k}_{1}}$$

19 $${k}_{\rm{app}}={k}_{1}\frac{{k}_{2}}{({k}_{2}+{k}_{-1})}$$

Multiple replicates of the experiment were conducted for TF–TMR (four replicates), CF–TMR (eight replicates) and CP–TMR (three replicates) and their corresponding CPY analogs (two replicates each). Results of the individual kinetic measurements are depicted in Supplementary Table 3. Replicates were averaged and the s.d. was calculated (Table 1 and Supplementary Table 4).

### Photophysical properties of labeled SNAP-tag2 and SNAP_f_-tag

For determination of the extinction coefficients (*ε*) of labeled SNAP-tag proteins, recombinant SNAP-tag2 and SNAP_f_-tag (30 µM) were labeled with fluorophore substrates (7.5 µM) in FP buffer overnight. For measurements, a serial dilution of the labeling reaction was performed, targeting final substrate concentrations of 7.5–2.22 µM. Absorbance spectra were recorded on a V-770 spectrophotometer (Jasco) in a quartz cuvette (3 mm). All measurements were performed in technical triplicates and absorbance values were baseline-corrected by setting the absorbance at 800 nm to zero using SpectraGryph version 1.2. For all fluorophores, the maximal absorbance value at each concentration was fitted to a linear function (Eq. (20)) and extinction coefficients were calculated from the slope *b* (Eq. (21)).

20 $$y(x)=a+{bx}$$

21 $$\varepsilon =\frac{b}{0.3}$$

Absolute QYs of fluorescently labeled SNAP-tag2 and SNAP_f_-tag were measured on a Quantaurs-QY spectrometer (model C11347, Hamamatsu). Measurements were carried out with diluted samples from the same reaction solution used for *ε* measurements (*A* = 0.03–0.08).

### Mammalian cell culture

All media were filtered before using sterile Nalgene Rapid-Flow filter units (0.22-μm pore size, 500 ml; Thermo Fisher Scientific). All experimental steps were performed under sterile conditions. Mammalian U2OS Flp In T-REx cells^50^ (U2OS cells) or Hela Kyoto Flp In cells^33^ (HeLa cells) were cultured in cell growth medium (DMEM + GlutaMAX supplemented with phenol red, high glucose (4.5 g l^−1^), pyruvate (Gibco Life Technologies, 31966-021) and 10% FBS (Gibco Life Technologies)) in either T-25 or T-75 culture flasks (Sarstedt). The cells were cultivated in a humidified incubator at 37 °C and 5% CO_2_ and passaged when reaching 80–90% confluency. Cell handling involved washing with PBS (1×, pH 7.4; Gibco Life Technologies, 10010-015), detachment (trypsin 0.05% + EDTA; TrypLE Express, Gibco Life Technologies, 12604-013), resuspension in DMEM + 10% FBS and transfer into fresh flasks. For imaging experiments, cells were handled in DMEM without phenol red, referred to as imaging medium (Gibco Life Technologies, 31053-028) supplemented with GlutaMAX (Gibco Life Technologies, 35050-038), pyruvate (Gibco Life Technologies, 11360-070) and 10% FBS (Gibco Life Technologies). For seeding cells at desired densities, cells were counted using a fluidlab R-300 cell counter (Anvajo) with Acella 100 chambers (20-μl sample volume; Anvajo). Cells were seeded in 96-well tissue culture plates (TPP, 92096) or 8-well or 96-well microplates with a glass bottom (Ibidi, 80827 and 89627, respectively) in a total volume of 100–150 μl of medium.

### Generation of mammalian stable cell lines

Stable cell lines were generated using the Flp In T-REx system (Thermo Fisher Scientific) with U2OS or HeLa cells. Cells were grown to ~80% confluency in T-25 flasks. Lipofectamine 3000 (8 μl; Thermo Fisher Scientific, L3000008) was diluted in Opti-MEM I reduced serum medium + GlutaMAX (200 μl; Thermo Fisher Scientific, 51985026) in a 1.5-ml reaction tube and thoroughly mixed. In a separate tube, the plasmid DNA encoding the gene of interest on a pcDNA/FRT or pcDNA/FRT/TO vector (440 ng) and pOG44 (3,560 ng; encoding the Flp In recombinase) were diluted in Opti-MEM I reduced serum medium + GlutaMAX (200 μl). P3000 (8.0 μl) was added to the DNA mix. After thorough mixing, the DNA–P3000 mix incubated at room temperature for 10 min. Subsequently, the DNA–P3000 mix was combined with the diluted Lipofectamine 3000, mixed and left to incubate at room temperature for an additional 15 min. The resulting DNA–P3000–Lipofectamine 3000 mix was added to the T-25 flask and incubated overnight. The following day, the medium was exchanged with cell growth medium supplemented with 100 μg ml^−1^ hygromycin B (Gibco Life Technologies), selecting cells for plasmid integration. Selection was performed for 48–72 h. Survived cells were recovered in cell growth medium to reach confluency. Cells were sorted using FACS for high expression levels according to a positive fluorescent protein signal (mEGFP, FITC filter) or SLP labeling (SiR, APC filter). Generated stable cell lines can be found in Supplementary Table 14.

### rAAV production and transduction

For dual-color STED imaging, recombinant adeno-associated viruses (rAAVs) were generated as described previously^51^. In brief, the AAV plasmid containing the CAG-Lifeact-HT7 expression cassette flanked by AAV2 packaging signals (inverted terminal repeats) were cotransfected with plasmids pRV1 (AAV2 Rep and Cap sequences), pH21 (AAV1 Rep and Cap sequences) and pFD6 (Adenovirus helper plasmid) using Lipofectamine 3000 into HEK293 cells. Then, 5 days after transfection, the cells were collected and lysed using TNT extraction buffer (20 mM Tris pH 7.5, 150 mM NaCl, 1% Triton X-100 and 10 mM MgCl_2_) for 10 min. The cell debris was removed by centrifugation (3,000*g*, 5 min, 4 °C), the cell supernatant was treated with Benzonase (4.5 µl, 30 min, 37 °C) and the rAAVs were purified by FPLC using HiTrap AVB Sepharose HP columns (Cytiva, 28411211). Purified rAAVs were concentrated using Amicon Ultra 15-ml (100-kDa MWCO) centrifugal filters (Merck) with buffer exchange to PBS pH 7.3.

U2OS Flp In T-REx cells stably expressing Vim–SNAP-tag2 were transduced with rAVVs 18 h before STED imaging by adding 0.5 μl of purified rAAVs (~10^9^–10^10^ rAAV particles) into 200 μl of imaging medium (DMEM GlutaMAX and 10% FBS, phenol red free; Gibco).

### Live-cell labeling performance of SNAP-tag proteins determined with flow cytometry

Flow cytometry was conducted on a BD Fortessa X-20 cell analyzer (BD Biosciences) equipped with a high-throughput screening (HTS) module for 96-well plates. Cells were seeded on transparent 96-well cell culture plates and treated according to desired experiment in a reaction volume of 100 µl. After treatment, labeling reaction was stopped by addition of recombinant SNAP-tag2 (2 µM, 100 µl, 10-min incubation), cells were washed twice with PBS (150 μl, 10-min incubation), trypsinized (50 μl of trypsin, 10-min incubation) and resuspended in FACS buffer (2% FBS in PBS) to a final volume of 200 μl. The cell suspension was transferred to nonbinding U-bottom 96-well plates (Falcon) and analyzed on a flow cytometer using the HTS module. Flow rates were set to 3 μl s^−1^ with three mixing steps and a subsequent washing volume of 400 μl. Cells were gated for live cells (SSC-A/FSC-A) and single cells (FSC-H/FSC-A) as exemplified in Supplementary Fig. 16. Excitation laser and emission filter settings were adjusted according to the fluorophore properties (Supplementary Table 9). PMT settings were adjusted to positive and negative controls. Data were analyzed using FlowJo software (BD Biosciences).

### Substrate screening for SNAP-tag labeling in live mammalian cells

U2OS Flp In T-REx (Thermo Fisher Scientific) cells stably expressing mEGFP–SNAP-tag or mEGFP–CLIP-tag fusion proteins were seeded into 96-well cell culture plates (10,000 cells per well) 1 day before the experiment. The cells were then incubated with substrates **1**–**30**, CP or BC (100 nM) for 2 h at 37 °C. All substrates were tested in technical triplicates. Cells were washed twice with cell growth medium for 150min incubation at 37 °C and once with sterile PBS pH 7.4 before detachment with trypsin (50 µl, 10 min, 37 °C). Cells were resuspended in FACS buffer to a final volume of 200 µl. The cell suspension was transferred to nonbinding U-bottom 96-well plates (Falcon) and analyzed on a flow cytometer using the HTS module, as previously described. Cells were gated for live-cell (SSC-A/FSC-A) and single-cell events (FSC-H/FSC-A) and for mEGFP expression signal (FITC channel) ≥ 10^3^. Gated cells were analyzed for their SLP labeling signal (TMR, PE channel) over expression level (mEGFP) and the medians of the ratios were derived using FlowJo software (BD Biosciences).

### Cell viability assay

U2OS cells were seeded on transparent 96-well cell culture plates 1 day before the experiment. Cells were incubated with TF–fluorophore, CF–fluorophore and CA–fluorophore substrates (1 µM) or DMSO (1% (v/v)) or remained untreated for 1 h at 37 °C. The medium was collected into nonbinding U-bottom 96-well plates (Falcon) and detached cells were harvested by centrifugation (3,000*g*, 5 min). The supernatant was removed. Additionally, adherent cells were detached with trypsin (30 µl, 10 min, 37 °C), resuspended in FACS buffer (2% FBS in PBS) to a final volume of 100 μl and added to the same wells to collect all dead and live cells. SYTOX blue dead cell stain (100 µl, 2 µM; Thermo Fisher Scientific) was added to the cells to a final concentration of 1 µM and cells were subsequently analyzed by flow cytometry (10,000 cells per well; laser: 405 nm, BP filter: 450/50). The experiment was performed in technical triplicates. Data were analyzed using FlowJo software (BD Biosciences).

### Confocal fluorescence microscopy of SNAP-tag2 in live mammalian cells

Confocal fluorescence microscopy of U2OS cells stably expressing HaloTag7–P30–SNAP-tag2/SNAP_f_-tag–NLS–P2A–NLS–mTurquoise2 was conducted on a Stellaris 5 microscope (Leica Microsystems). Live-cell imaging was performed in 8-well or 96-well glass bottom dishes (Ibidi) at 37 °C and 5% CO_2_ in a humidified chamber. Images were acquired with a ×40 (HC PL APO CS2 ×40/1.10) water objective, a scan speed of 600 Hz, line average of 2, optical zoom of 1–1.3, image size 1,024 × 1,024 μm and 12-bit pixel depth. *Z* stacks were recorded with a step size of 2–5 μm. HaloTag7 and SNAP_f_-tag/SNAP-tag2 were labeled with their respective TF–fluorophore or CA–fluorophore substrates at 100 nM overnight. Cells were washed twice with PBS before imaging in imaging medium. Laser settings are described in Supplementary Table 10. Image analysis was performed using Fiji software^52^. For visualization purposes, maximum intensity projections of *z*-stacked images were calculated. For quantification, intensities of multiplane images were summed and regions of interest (ROIs) were defined manually. Mean fluorescence intensities of the ROI’s fluorescence channels were calculated (multi-ROI measurement) and ratios calculated from fluorescent labels to respective expression signals were compared. Ratiometric projections were generated using the BRET-analyzer plugin^53^.

### In-cell kinetic measurements of SNAP-tag2 and SNAP_f_-tag

In-cell kinetic measurements of SNAP-tag2 and SNAP_f_-tag labeling with TF–fluorophore, CF–fluorophore and CP–fluorophore substrates were performed in U2OS cells stably expressing HaloTag7–P30–SNAP-tag2/SNAP_f_-tag–NLS–P2A–NLS–mTurquoise2 on a Stellaris 5 microscope as previously described. Fluorophore substrates were added at a final concentration of 50 nM (for TMR and CPY) or 100 nM (for SiR) to the cells and images with three *z* stacks were acquired every 30 s over time. For quantification, fluorescence intensities of multiplane images were summed and the ratios of fluorophore substrate signals normalized to mTurquoise2 expression signals were analyzed using CellProfiler^54^. Additional image acquisition parameters were as follows: ×20 water objective (HC PL APO CS2 ×20/0.75 IMM), ×1.28 optical zoom, image size 1,024 × 1,024 μm, scan speed of 400 Hz and line average of 2. Laser settings are described in Supplementary Table 11. Kinetic data were analyzed by fitting a sigmoidal curve (Eq. (22)) in GraphPad Prism 10.2.3.

22 $$Y={Y}_{0}+\frac{({Y}_{\max }-{Y}_{0})}{\left(1+{\left(\frac{{t}_{1/2}}{x}\right)}^{H}\right)}$$

where *Y* is the fluorescence intensity ratio (signal/mTurquoise2), *Y*_max_ is the plateau of the fluorescence intensity ratio, *Y*_0_ is the *y*-intercept, *H* is the Hill slope and *x* is time (min).

The experiment was conducted in biological duplicates and the average *t*_1/2_ of both replicates was calculated. Respective CIs were propagated by Eqs. (23–26) as follows:

1. Calculate the mean *t*_1/2_ of both replicates:
  23 $$\overline{{t}_{1/2}}=\frac{{({t}_{1/2})}_{1}+{({t}_{1/2})}_{2}}{2}$$
2. Compute the standard error (SE) for each *t*_1/2_ of a replicate:
  24 $$\begin{array}{c}{{\rm{SE}}}_{{({t}_{1/2})}_{1}}=\frac{{\rm{CI}}_{{1}_{\rm{high}}}-{\rm{CI}}_{{1}_{\rm{low}}}}{2\times 1.96}\\ {{\rm{SE}}}_{{({t}_{1/2})}_{2}}=\frac{{\rm{CI}}_{{2}_{\rm{high}}}-{\rm{CI}}_{{2}_{\rm{low}}}}{2\times 1.96}\end{array}$$
3. Calculate the s.e.m.:
  25 $${\rm{SE}}_{\overline{({t}_{1/2})}}=\sqrt{\frac{{{{\rm{SE}}}^{2}}_{{({t}_{1/2})}_{1}}+{{{\rm{SE}}}^{2}}_{{({t}_{1/2})}_{2}}}{2}}$$
4. Compute the 95% CI for the average *t*_1/2_:

26 $${\rm{CI}}_{\overline{({t}_{1/2})}}=\overline{{t}_{1/2}}\pm 1.96\times {\rm{SE}}_{\overline{({t}_{1/2})}}$$

where $\overline{{t}_{1/2}}$ is the *t*_1/2_ of the duplicate, CI_high_ is the higher 95% CI and CI_low_ is the lower 95% CI.

### Live-cell STED microscopy in mammalian cells

HeLa cells stably expressing Cox8a–SLP fusion proteins together with mEGFP were labeled with respective CA–SiR or TF–SiR (100 nM) substrates for 1 h and washed twice with imaging medium before microscopy. U2OS cells stably expressing Vim–SNAP_f_-tag, Vim–SNAP-tag2, TOMM20–SNAP-tag2 or CalR–SNAP-tag2-KDEL or coexpressing Vim–SNAP-tag2 and LifeAct–HaloTag7 (rAAV) were labeled with CF–SiR and CA–MaP618 (100 nM), respectively, for 1 h and washed twice with imaging medium before microscopy. Live-cell STED nanoscopy was performed using an Abberior STED Expert Line 595/775/RESOLFT QUAD scanning microscope (Abberior Instruments). SiR was excited using a 640-nm excitation line and depleted using a 775-nm STED line. For dual-color imaging, MaP618 was excited with a 561-nm excitation line and depleted with the same STED line. The microscope was equipped with a UPlanSApo ×100/1.4 oil immersion objective lens (Olympus) and the fluorescence signal was detected using avalanche photodiodes. The pinhole was set to 0.9 Airy units, a gating of 0.75–8 ns was applied and dwell times of 7–10 µs and a pixel size of 25 nm were used. For STED images, each line was scanned 4–8 times and the signal was accumulated. Imaging settings are further detailed in Supplementary Table 12. Lookup tables used for image representation in Fig. 4a–e were as follows: green, mEGFP; red-hot, SiR; cyan, SNAP-tag2–SiR; orange-hot, HaloTag7–MaP618. For image representation, a Gaussian blur filter with a 2*σ* radius was applied.

The FWHM of single-intermediate filaments (Vim–SNAP_f_-tag/SNAP-tag2 fusion) was determined by extracting fluorescence intensity profiles perpendicular to single filaments using Fiji software (ImageJ 1.53t)^52^. Mean filament diameters were calculated from 15 individual fibrils and from three individual images (*n* = 3) by fitting a Gaussian function (Eq. (27)), yielding the FWHM from Eq. (28).

27 $$y={y}_{0}+\frac{A}{\omega \sqrt{\frac{\pi }{2}}}{e}^{-2\frac{{(x-{x}_{c})}^{2}}{{\omega }^{2}}}$$

where *y* is the normalized fluorescence intensity, *x* is the *x* coordinate (μm), *y*_0_ is the offset, *A* is the area, *ω* is the width and *x*_c_ is the center.

28 $${\rm{FWHM}}=\omega \sqrt{2\mathrm{ln}(2)}$$

where *ω* is the Gauss width.

STED microscopy images in Fig. 4a are snapshots of three independent experiments (eight images in total) with similar outcome. Additionally, five individual experiments (12 images in total) with labeling substrate concentrations ranging from 25 to 250 nM and incubation times ranging from 1 h to overnight labeling in two different cell types (U2OS and HeLa cells) yielded similar brightness differences. Images shown in Fig. 4b are snapshots of two individual experiments (seven images in total). FWHM quantification was conducted for one individual experiment (15 filaments, three images). STED images in Fig. 4c–e were acquired in one individual experiment.

### Strain and growth conditions of *H.polymorpha* yeast cells

*H.* *polymorpha* ku80 yeast cells were grown at 37 °C with shaking (200 rpm). For fluorescence microscopy studies, the cultures were grown in mineral medium (MM)^55^ containing 30 µg ml^−1^ leucine, 1× vitamin solution, 0.25% ammonium sulfate and 0.5% methanol.

### Construction of *H.polymorpha* strains expressing Pex3–SNAP-tag2/SNAP_f_-tag

The SNAP_f_-tag DNA sequence was synthesized by Genscript and SNAP-tag2 was PCR-amplified from pET51b-SNAP-tag2 using primers Fw_BglII_SNAP-tag2 (GATGCTAGATCTGATAAAGACTGCGAAATGAAACG) and Rv_SalI_SNAP-tag2 (CTCTAGAGTCGACCTAGCCCAGGCCTGGTTTACCC). Both SNAP-tag inserts were cloned into the pUC57 plasmid vector. pHIPZ_Pex3_mGFP^56^ and pUC57_SNAP_f_-tag/SNAP-tag2 were restricted using SalI and BglII according to the supplier protocol (Thermo Fisher Scientific) and ligated to generate pHIPZ_Pex3_SNAP_f_-tag or pHIPZ_Pex3_SNAP-tag2 plasmids for peroxisome targeting in yeast. Plasmids were transformed into *E.* *coli* DH5α cells selected on LB with ampicillin. Positive colonies were checked using XhoI restriction. The plasmids were linearized with EcoRI or MluI and transformed into *H.* *polymorpha* yku80 cells by electroporation as described in Faber et al.^57^. Positive integrations in yeast were tested with colony PCR using primers Ppex3_Fw (CCTGTTGCGGCAAGATATAG) and Seq_SNAP_Rv (CGTAGGAAGGCTGGATGTC). Plasmids were confirmed by LightRun sequencing (Eurofins Genomics) and compared using Clone Manager 9 (Scientific and Educational Software). Correct yeast integration was checked by colony PCR with DreamTaq enzyme (Thermo Fisher Scientific) of zymolyase-treated cells and imaged using the Gel Doc XR+ System (Bio-Rad).

### Live-cell labeling of yeast peroxisomes

*H.* *polymorpha* cells were grown until stationary phase in MM supplemented with 0.5% glucose at 37 °C shaking (200 rpm). The culture was diluted to OD_660_ = 0.1 and grown until OD_660_ > 1.6. This culture was again diluted to OD_660_ = 0.1 in MM and methanol and the cells were simultaneously stained using TF–SiR, CP–SiR, BG–SiR, TF–MaP555, CP–MaP555 or BG–MaP555 at a final concentration of 250 nM for 18 h at 37 °C shaking, reaching a final OD_660_ of ~3.0. To stain the yeast cell walls, cells were incubated with 25 µg ml^−1^ Calcofluor white (Fluorescent Brightner 28; Sigma-Aldrich, 910090) for 15 min at 37 °C shaking. Cells were washed three times with PBS and immediately used for live-cell imaging in MM and methanol.

### Live-cell STED microscopy of yeast peroxisomes

STED nanoscopy was conducted on a commercial microscope (Abberior Instruments) equipped with a STED depletion laser (775 nm), four excitation lasers (640, 561, 488 and 405 nm), a CoolLED pE-2 excitation system and a ×100 oil immersion objective (Olympus UPLSAPO/1.40). Confocal overview images (80 × 80 µm) were taken using a pixel size of 180 nm, dwell time of 20 µs and 6% laser power using the 561-nm excitation laser with 570–680-nm detection for MaP555 substrates or 0.5% laser power using the 640-nm excitation laser with 650–730-nm detection for SiR substrates. STED images were taken with a pixel size of 25 nm, dwell time of 20 µs and 30% laser power using the 561-nm excitation laser with 570–680-nm detection for MaP555 substrates or 2% laser power using the 640-nm excitation laser with 650–730-nm detection for SiR substrates. A depletion laser power of 50% or 10% at 775 nm was applied for 561-nm or 640-nm excitation, respectively. Calcofluor white was imaged using 3% laser power with the 405-nm excitation laser and 410–480-nm detection. Image acquisition was carried out using Imspector software (version 16.3, Abberior Instruments). Analysis was performed using Fiji software (ImageJ 2.14.014)^52^. To compare the fluorescence intensities of SNAP_f_-tag and SNAP-tag2 labeled with different fluorophore substrates, the mean and maximum fluorescence intensity of 125 cells (rectangles of 3 × 3 µm as the ROI) were measured in biological triplicates. Line profile measurements were performed for one cell per condition.

### Statistics and reproducibility

All in vitro measurements were performed at least in technical triplicates. Flow cytometry-based experiments were performed at least in technical duplicates. Live-cell kinetic measurements were performed as biological duplicates (that is, on two different days 1 week apart). STED images aiming to compare differences in labeling brightness or resolution in mammalian cells were repeated at least two times to ensure similar outcomes. Quantification of fluorescence intensities in yeast cells were performed in biological triplicates. Unless otherwise stated, STED images originated from single experiments. Statistical significance of a sample group over a reference group was performed by performing a two-tailed unpaired *t*-test with Welch’s correction using GraphPad Prism (version 10.2.3). Sample sizes are indicated in the captions of corresponding figures. The following notations apply for all statistical analyses: NS (not significant), *P* ≥ 0.05, *P < 0.05, **P < 0.01, ***P < 0.001 and ****P < 0.0001.

### Reporting summary

Further information on research design is available in the Nature Portfolio Reporting Summary linked to this article.

## Online content

Any methods, additional references, Nature Portfolio reporting summaries, source data, extended data, supplementary information, acknowledgements, peer review information; details of author contributions and competing interests; and statements of data and code availability are available at 10.1038/s41589-025-01942-z.

## Supplementary information

Supplementary Information Supplementary Figs. 1–143 and Tables 1–19, protein sequences, scripts and chemical synthesis procedures.

Reporting Summary

Supplementary Data 1 Source data for supplementary figures.

## Source data

Source Data Fig. 2 Statistical source data.

Source Data Fig. 3 Statistical source data.

Source Data Fig. 5 Statistical source data.

Source Data Extended Data Fig. 2 Statistical source data.

Source Data Extended Data Fig. 3 Statistical source data.

Source Data Extended Data Fig. 4 Statistical source data.

Source Data Extended Data Fig. 6 Statistical source data.

## Data Availability

Plasmids of interest from the study are available on Addgene. Addgene accession codes can be found in Supplementary Table 14. The data supporting the findings of this study are available within the paper and its Supplementary Information. Data are available from the corresponding authors upon request. Source data are provided with this paper.

## Code availability

All code and example scripts used in this study are available within the manuscript and its Supplementary Information.
