# Supplementary material for: SNAP-tag2 for faster and brighter protein labeling
Source: Nat Chem Biol. 2025 Jul 3;21(11):1754–61. doi: 10.1038/s41589-025-01942-z (PMC12568630; doi:10.1038/s41589-025-01942-z)
Supplement: Supplementary file 2 — Reporting Summary [file 41589_2025_1942_MOESM2_ESM.pdf]

nature portfolio

Corresponding author(s): Dr. Julien Hiblot, Prof. Dr. Kai Johnsson

Last updated by author(s): Mar 10, 2025

Reporting Summary

Nature Portfolio wishes to improve the reproducibility of the work that we publish. This form provides structure for consistency and transparency in reporting. For further information on Nature Portfolio policies, see our Editorial Policies and the Editorial Policy Checklist.

Statistics

For all statistical analyses, confirm that the following items are present in the figure legend, table legend, main text, or Methods section.

n/a	Confirmed	
☐	☒	The exact sample size (*n*) for each experimental group/condition, given as a discrete number and unit of measurement
☐	☒	A statement on whether measurements were taken from distinct samples or whether the same sample was measured repeatedly
☐	☒	The statistical test(s) used AND whether they are one- or two-sided<br>*Only common tests should be described solely by name; describe more complex techniques in the Methods section.*
☒	☐	A description of all covariates tested
☐	☒	A description of any assumptions or corrections, such as tests of normality and adjustment for multiple comparisons
☐	☒	A full description of the statistical parameters including central tendency (e.g. means) or other basic estimates (e.g. regression coefficient) AND variation (e.g. standard deviation) or associated estimates of uncertainty (e.g. confidence intervals)
☐	☒	For null hypothesis testing, the test statistic (e.g. *F*, *t*, *r*) with confidence intervals, effect sizes, degrees of freedom and *P* value noted<br>*Give P values as exact values whenever suitable.*
☒	☐	For Bayesian analysis, information on the choice of priors and Markov chain Monte Carlo settings
☒	☐	For hierarchical and complex designs, identification of the appropriate level for tests and full reporting of outcomes
☒	☐	Estimates of effect sizes (e.g. Cohen's *d*, Pearson's *r*), indicating how they were calculated

Our web collection on statistics for biologists contains articles on many of the points above.

Software and code

Policy information about availability of computer code

Data collection	Plate reader: Tecan Sparkcontrol Method Editor Version 2.2<br>Stopped-flow device: BioLogic SFM-400<br>Confocal Microscopy: Leica Stellaris 5<br>STED Microscopy: Abberior STED Expert Line 595/775/RESOLFT QUAD scanning microscope<br>FACS: BD FACSMelody™ Cell Sorter<br>Flow cytometry: BD Fortessa™ X-20 Cell Analyzer<br>NMR: BRUKER Advance III HD 400 NMR spectrometer equipped with a CryoProbe™<br>Thermal stability: Prometheus NT48 nanoscale differential scanning fluorimeter<br>Photophysical properties: Quantaurs-QY spectrometer (model C11347, Hamamatsu), V-770 Spectrophotometer (Jasco)<br>Chemical property calculation: Schroedinger Maestro 12.3
Data analysis	General data analysis: GraphPad Prism (version 10.2.3), DynaFit 4 (version 4.11.050), SpectraGryph (version 1.2), R Studio (version 4.3.1),<br>Molecular Biology: NEBaseChanger (version 1.3.3, nebasechanger.neb.com), Tm Calculator (version 1.15.0, tmcalculator.neb.com), Geneious Prime (version 2023.1.2)<br>Image analysis: ImageJ 1.54f, Leica LAS X 3.5.7.23225 (Confocal), CellProfiler (version 4.2.6), Inspector (version 16.3, Abberior Instruments)<br>Chemistry: MestReNova (version 14.1.0-24037), ChemDraw (version 20.1.1)<br>Crystal structures: PyMOL version 2.1.1.

For manuscripts utilizing custom algorithms or software that are central to the research but not yet described in published literature, software must be made available to editors and reviewers. We strongly encourage code deposition in a community repository (e.g. GitHub). See the Nature Portfolio guidelines for submitting code & software for further information.

Data

Policy information about availability of data

All manuscripts must include a data availability statement. This statement should provide the following information, where applicable:
- Accession codes, unique identifiers, or web links for publicly available datasets
- A description of any restrictions on data availability
- For clinical datasets or third party data, please ensure that the statement adheres to our policy

Plasmids encoding certain SNAP-tag2 constructs have been deposited on Addgene. Addgene numbers can be found in Supplementary Table 14. Correspondence and requests for materials should be addressed to Kai Johnsson. The data supporting the main findings of this study are available within the article and its Supplementary Information and are available from the corresponding author upon request. Source data are provided with this manuscript.
The crystal structures of SNAP-tag in its apo (PDB: 3KZY), benzylated (PDB: 3L00), BG-bound (PDB: 3KZZ) and TMR-labeled (PDB: 6Y8P) states were available from the pdb.

Human research participants

Policy information about studies involving human research participants and Sex and Gender in Research.

Reporting on sex and gender	Sex and gender was not necessary for the presented experiments, as the data is not relevant to a clinical trial.
Population characteristics	Population characteristics was not necessary for the presented experiments, as the data is not relevant to a clinical trial.
Recruitment	Recruitment was not necessary for the presented experiments, as the data is not relevant to a clinical trial.
Ethics oversight	Ethics oversight was not necessary for the presented experiments, as the data is not relevant to a clinical trial.

Note that full information on the approval of the study protocol must also be provided in the manuscript.

Field-specific reporting

Please select the one below that is the best fit for your research. If you are not sure, read the appropriate sections before making your selection.

☒ Life sciences ☐ Behavioural & social sciences ☐ Ecological, evolutionary & environmental sciences

For a reference copy of the document with all sections, see nature.com/documents/nr-reporting-summary-flat.pdf

Life sciences study design

All studies must disclose on these points even when the disclosure is negative.

Sample size	Samples were acquired until a clear trend was evident.
Data exclusions	No data was excluded.
Replication	In vitro measurements were performed in technical triplicates or as indicated. Microscopy experiments were performed on one to three independent sample preparations as stated in the method section. All replicates were successful.
Randomization	Randomization is not relevant to the study, as all experiments were performed with cell lines. No experiments involved allocation of different samples, organism, or participants into experimental groups.
Blinding	Blinding is not relevant to the study because no experiments involved allocation of different samples, organisms, or participants into experimental groups.

Reporting for specific materials, systems and methods

We require information from authors about some types of materials, experimental systems and methods used in many studies. Here, indicate whether each material, system or method listed is relevant to your study. If you are not sure if a list item applies to your research, read the appropriate section before selecting a response.

Materials & experimental systems

n/a	Involved in the study
☐	☒ Antibodies
☐	☒ Eukaryotic cell lines
☒	☐ Palaeontology and archaeology
☒	☐ Animals and other organisms
☒	☐ Clinical data
☒	☐ Dual use research of concern

Methods

n/a	Involved in the study
☒	☐ ChIP-seq
☐	☒ Flow cytometry
☒	☐ MRI-based neuroimaging

Antibodies

Antibodies used

primary Anti-c-Myc (Ab-1) Mouse mAb (9E10) (#OP10, EMD Millipore, Merck),
secondary goat anti-mouse-Alexa647 antibody (#A-21236, Invitrogen, ThermoFisher Scientific)

Validation

Anti-c-Myc (Ab-1) Mouse mAb (9E10) is validated for use in FC, Frozen Sections, Immunoblotting, IF, IP, Chromatin IP, Paraffin Sections for the detection of c-Myc (Ab-1) as stated by the manufacturer (https://www.merckmillipore.com/TH/en/product/Anti-c-Myc-Ab-1-Mouse-mAb-9E10,EMD_BIO-OP10?ReferrerURL=https%3A%2F%2Fwww.google.com%2F#documentation).

Eukaryotic cell lines

Policy information about cell lines and Sex and Gender in Research

Cell line source(s)

U-2 OS Flp-In T-REx cell lines (Molecular and Cellular Biology 2006, 26 (12), 4642-4651) - from Blacklow lab. Department of Pathology, Brigham and Women's Hospital and Harvard Medical School, Boston, MA 02115, USA.
HeLa Kyoto Flp-In cells (kind gift of Dr. Amparo Andres-Pons (EMBL, Heidelberg); Science 2024, 383, 890–897)

Authentication

Cell lines were not further authenticated.

Mycoplasma contamination

Cell lines have been tested by PCR and were negative.

Commonly misidentified lines
(See ICLAC register)

Not applicable as no commonly misidentified cell lines were used.

Flow Cytometry

Plots

Confirm that:

☒ The axis labels state the marker and fluorochrome used (e.g. CD4-FITC).

☒ The axis scales are clearly visible. Include numbers along axes only for bottom left plot of group (a 'group' is an analysis of identical markers).

☒ All plots are contour plots with outliers or pseudocolor plots.

☒ A numerical value for number of cells or percentage (with statistics) is provided.

Methodology

Sample preparation

Protein labeling on yeast surface for fluorescence-activated cell sorting (FACS). For fluorescent labeling, 10^7 cells were harvested by centrifugation (14’000×g, 1 min.). For antibody-based expression staining of yeast cells transformed with pCTcon2 plasmids, yeast cells were resuspended in 1:10-diluted primary mouse anti-cMyc antibody (#OP10, EMD Millipore, Merck) in PBS (50 µL) and incubated on a rotating wheel at 4 °C for 1 h. The cells were pelleted (14’000×g, 1 min.) and washed twice with PBS (125 µL) with centrifugation in-between (14’000×g, 1 min.). The cell pellet was resuspended in 1:50-diluted secondary goat anti-mouse-Alexa647 antibody (#A-21236, Invi-trogen, ThermoFisher Scientific) in PBS (50 µL) and incubated on a rotating wheel at 4 °C for 1 h. The cells were washed twice with PBS (125 µL) prior to labeling with SNAP-tag substrates. For protein libraries encoded by the pJYDNg expression vector, expression control was monitored by labeling of eUnaG2 with bilirubin. Yeast cells were resuspended in PBS (50 µL) containing bilirubin [10 µM] and BSA [1 mg/mL], gently vortexed and incubated on ice for 10 min. The cells were pelleted and washed twice with PBS (150 µL) interspaced by centrifugation steps (14’000×g, 1 min.) prior to SNAP-tag labeling. Cells expressing SNAP-tag variants were labeled in PBS (50 µL) using different substrates (CF3P-TMR/-MaP618, TF-TMR/-MaP618) with varying concentrations [10-500nM] and incubation times (10-60 min) in order to adjust the screening stringency. Labeling was performed at r.t. on a rotating wheel. Cells were washed with PBS (125 µL), resuspended in 1 mL of PBS and filtered through 5 mL round bottom polystyrene test tubes with cell strainer snap caps (#352235, Falcon®) for FACS.

Live cell labeling performance of SNAP-tag proteins determined with flowcytometry. Cells were seeded on transparent 96-well cell culture plates and treated according to desired experiment in a reaction volume of 100 µL. After treatment, labeling reaction was stopped by addition of recombinant SNAP-tag2 (2 µM, 100 µL, 10 min. incubation), cells were washed twice with PBS (150 µL, 10 min. incubation), trypsinized (50 µL trypsin, 10 min. incubation) and resuspended in FACS buffer (2 %

FBS in PBS) to a final volume of 200 µL. The cell suspension was transferred to non-binding u-bottom 96-well plates (Falcon) and analyzed on the flow cytometer using the HTS module.

Substrate screening for SNAP-tag labeling in live mammalian cells. U2OS Flp In™ T-REx™ (Thermo Fisher) cells stably expressing mEGFP-SNAP-tag or mEGFP-CLIP-tag fusion proteins were seeded into 96-well cell culture plates (10’000 cells/well) the day prior to experiment. The cells were then incubated with substrates 1 – 30, CP or BC [100 nM] for 2 h at 37 °C. All substrates were tested in technical triplicates. Cells were washed twice with cell growth medium for 15 min incubation at 37°°C and 1x with sterile PBS (pH = 7.4) prior to detachment with trypsin (50 µL, 10 min., 37 °C). Cells were resuspended in FACS buffer to a final volume of 200 µL. The cell suspension was transferred to non-binding u-bottom 96-well plates (Falcon) and analyzed on the flow cytometer using the HTS module.

Cell viability assay. U2OS cells were seeded on transparent 96-well cell culture plates one day prior to experiment. Cells were incubated with TF-, CF- and CA-fluorophore substrates [1 µM], DMSO [1 % (v/v)] or remained untreated for 1 h at 37 °C. The medium was collected into non-binding u-bottom 96-well plates (Falcon) and detached cells were harvested by centrifugation (3000 x g, 5 min). The supernatant was removed. Additionally, adherent cells were detached with trypsin (30 µL, 10 min., 37 °C), resuspended in FACS buffer (2 % FBS in PBS) to a final volume of 100 µL and added to the same wells to collect all dead and live cells. SYTOX Blue dead cell stain (100 µL, 2 µM; ThermoFisher) was added to the cells to a final concentration of 1 µM and cells were subsequently analyzed by flow cytometry (10’000 cells/well; laser: 405 nm, BP filter: 450/50).

Instrument

BD Fortessa™ X-20 Cell Analyzer, BD FACSMelody™ Cell Sorter

Software

FlowJo (version 10.10.0)

Cell population abundance

For yeast: the final sorted population was 0.5-3% of all cell events
For mammalian cells: the final sorted population was up to 10-50% of all cell events.

Gating strategy

Hierarchical gating of live cells (SSC-A/FSC-A), single cells (FSC-H/FSC-A) and self-labeling protein labeling (label channel/expression channel). Gating strategy is described in the method section and exemplified in Supplementary Figures 15 and 16.

☒ Tick this box to confirm that a figure exemplifying the gating strategy is provided in the Supplementary Information.
